# Drug Discovery Using Chemical Systems Biology: Repositioning the Safe Medicine Comtan to Treat Multi-Drug and Extensively Drug Resistant Tuberculosis

**DOI:** 10.1371/journal.pcbi.1000423

**Published:** 2009-07-03

**Authors:** Sarah L. Kinnings, Nina Liu, Nancy Buchmeier, Peter J. Tonge, Lei Xie, Philip E. Bourne

**Affiliations:** 1 Department of Biology, University of York, York, United Kingdom; 2 Institute of Chemical Biology & Drug Discovery, Department of Chemistry, Stony Brook University, Stony Brook, New York, United States of America; 3 Department of Chemistry and Biochemistry, University of California San Diego, La Jolla, California, United States of America; 4 San Diego Supercomputer Center, University of California San Diego, La Jolla, California, United States of America; 5 Skaggs School of Pharmacy and Pharmaceutical Sciences, University of California San Diego, La Jolla, California, United States of America; University of Houston, United States of America

## Abstract

The rise of multi-drug resistant (MDR) and extensively drug resistant (XDR) tuberculosis around the world, including in industrialized nations, poses a great threat to human health and defines a need to develop new, effective and inexpensive anti-tubercular agents. Previously we developed a chemical systems biology approach to identify off-targets of major pharmaceuticals on a proteome-wide scale. In this paper we further demonstrate the value of this approach through the discovery that existing commercially available drugs, prescribed for the treatment of Parkinson's disease, have the potential to treat MDR and XDR tuberculosis. These drugs, entacapone and tolcapone, are predicted to bind to the enzyme InhA and directly inhibit substrate binding. The prediction is validated by *in vitro* and InhA kinetic assays using tablets of Comtan, whose active component is entacapone. The minimal inhibition concentration (MIC_99_) of entacapone for *Mycobacterium tuberculosis* (*M.tuberculosis*) is approximately 260.0 µM, well below the toxicity concentration determined by an *in vitro* cytotoxicity model using a human neuroblastoma cell line. Moreover, kinetic assays indicate that Comtan inhibits InhA activity by 47.0% at an entacapone concentration of approximately 80 µM. Thus the active component in Comtan represents a promising lead compound for developing a new class of anti-tubercular therapeutics with excellent safety profiles. More generally, the protocol described in this paper can be included in a drug discovery pipeline in an effort to discover novel drug leads with desired safety profiles, and therefore accelerate the development of new drugs.

## Author Summary

The rise of multi-drug resistant (MDR) and extensively drug resistant (XDR) tuberculosis around the world, including in industrialized nations, poses a great threat to human health. This resistance highlights the need to develop new, effective and inexpensive anti-tubercular agents. Unfortunately, conventional approaches have yielded very few successes in the field of anti-infective drug discovery. It is a challenge to design drugs with both efficacy and safety. These challenges are reflected in the high costs involved in bringing new drugs to market. It has been estimated that the cost to launch a successful new drug is in excess of US$800 million. We have developed a novel computational strategy to systematically identify cross-reactivity between different drug target families. In this paper we demonstrate the strength of this approach through the discovery that existing commercially available drugs prescribed for the treatment of Parkinson's disease have the potential to treat MDR and XDR tuberculosis. The protocol described herein can be included in a drug discovery pipeline in an effort to accelerate the development of new drugs with reduced side effects.

## Introduction

Tuberculosis, which is caused by the bacterial pathogen *Mycobacterium tuberculosis* (*M.tuberculosis*), is a leading cause of mortality among infectious diseases. It has been estimated by the World Health Organization (WHO) that almost one-third of the world's population, around 2 billion people, is infected with the disease [1]. Every year, more than 8 million people develop an active form of the disease, which subsequently claims the lives of nearly 2 million. This translates to over 4,900 deaths per day, and more than 95% of these are in developing countries [2]. In 2002, the WHO estimated that if the worldwide spread of tuberculosis was left unchecked, then the disease would be responsible for approximately 36 million more deaths by the year 2020.

Despite the current global situation, anti-tubercular drugs have remained largely unchanged over the last four decades [3]. The widespread use of these agents, and the time needed to remove infection, has promoted the emergence of resistant *M.tuberculosis* strains. Multi-drug resistant tuberculosis (MDR-TB) is defined as resistance to the first-line drugs isoniazid and rifampin. The effective treatment of MDR-TB necessitates the long-term use of second-line drug combinations, an unfortunate consequence of which is the emergence of extensively drug resistant tuberculosis (XDR-TB) – *M.tuberculosis* strains that are resistant to isoniazid plus rifampin, as well as key second-line drugs, such as ciprofloxacin and moxifloxacin. XDR-TB is extremely difficult to treat because the only remaining drug classes exhibit very low potency and high toxicity. The rise of XDR-TB around the world, including in industrialized nations, imposes a great threat on human health, therefore emphasizing the need to identify new anti-tubercular agents as an urgent priority [4].

Currently, anti-infective therapeutics are discovered and developed by either *de novo* strategies, or through the extension of available chemical compounds that target protein families with the same or similar structures and functions. *De novo* drug discovery involves the use of high throughput screening techniques to identify new compounds, both synthetic and natural, as novel drugs. Unfortunately, this approach has yielded very few successes in the field of anti-infective drug discovery [5]. Indeed, the progression from early-stage biochemical hits to robust lead compounds is commonly an unfruitful process. The identification of both molecular targets that are essential for the survival of the pathogen, and compounds that are active on intact cells, is a challenging task. Even more formidable, however, is the requirement for appropriate potency levels and suitable pharmacokinetics, in order to achieve efficacy in small animal disease models [5]. These challenges are reflected in the high costs involved in bringing new drugs to market. In fact, it has been estimated that the successful launch of a single new drug costs more than US$800 million [6].

Two alternative drug discovery strategies that circumvent some of the challenges associated with *de novo* drug discovery are the label extension and ‘piggy-back’ strategies, both of which are widely employed for the discovery of novel therapeutics to treat tropical diseases. Label extension is a fast-track approach that involves the extension of the indications of an existing treatment to another disease. Some of the most important anti-parasitic drugs in use today, such as praziquantel for schistosomiasis, were derived from the label extension process. The major advantages of label extension are the significant reductions in cost and time to market that can be achieved. Alternatively, when a molecular target that is present in a pathogen is under investigation for other commercial indications, it is possible to adopt the ‘piggy-back’ strategy by utilizing the identified chemical starting points. Examples of this approach include the anti-malarial screening of a lead series of cysteine protease inhibitors for the treatment of osteoporosis, and histone deacetylase inhibitors for use in cancer chemotherapy [5].

One of the main aims of drug discovery is to develop safe and effective therapeutic agents through the optimization of binding to a specific protein target. In this way, undesirable effects resulting from side scatter pharmacology are minimized. However, the recent and rapid completion of numerous genome sequencing projects has revealed that proteins involved in entirely different biochemical pathways, and even residing in different tissues and organs, may possess functional binding pockets with similar shapes and physiochemical properties [7]. Therefore, chemical matter for one target could be considered as the basis for leads for an entirely different target. Recent work on large scale mapping of polypharmacology interactions by Paolini *et al.* [8] revealed the extent of promiscuity of drugs and leads across the proteome. They discovered that around 35% of 276,122 active compounds in their database had observed activity for more than one target. Whilst the majority of these promiscuous compounds were found to be active against targets within the same gene family, a significant number (around 25%) had recorded activity across different gene families.

The finding that so many drugs interact with more than one target provided the rationale behind the selective optimization of side activities (SOSA) approach recently developed by Wermuth [9],[10]. The SOSA approach involves the use of old drugs for new pharmacological targets, which is a valuable concept considering the finite number of small molecules that can be safely administered to humans. The process itself involves screening a limited number of structurally diverse drug molecules, and then optimizing the hits so that they show a stronger affinity for the new target and a weaker affinity for the original target(s). In this way, it is possible to derive a whole panel of new active molecules from a single marketed drug. Since the screened drug molecules already have known safety and bioavailability in humans, the overall time and cost of drug discovery is significantly reduced when compared with *de novo* strategies.

We have developed a novel computational strategy to identify off-targets of major pharmaceuticals on a proteome-wide scale [11]–[15]. Our methodology extends the scope of the SOSA concept effectively and systematically across gene families, and is more likely to be successful in achieving the ultimate goal of providing new drugs from old ones. Our chemical systems biology approach proceeds as follows:

1. The binding site of a commercially available drug is extracted or predicted from a 3D structure or model of the target protein [13].
2. Off-targets with similar ligand binding sites are identified across the proteome using an efficient and accurate functional site search algorithm [11].
3. Atomic interactions between the putative off-targets and the drug are evaluated using protein-ligand docking. Only those off-targets that do not experience serious atomic clashes with the drug are selected for further analysis.
4. The drug is further optimized to enhance its potency, selectivity and ADME properties by taking into account both the primary target and the off-targets across the genome.

Our approach essentially explores complex protein-ligand interaction networks on a proteome-wide scale. The lead compound can be discovered from all drug targets across different gene families. Moreover, lead optimization can focus on compounds with excellent safety profiles and known clinical outcomes. In this way, our approach has the potential to increase the rate of successful drug discovery and development, whilst reducing the costs involved.

In the present study we demonstrate the efficiency and efficacy of our chemical systems biology approach through the discovery of safe chemical compounds with the potential to treat MDR-TB and XDR-TB. The identified compounds are entacapone and tolcapone. These drugs primarily target human catechol-*O*-methyltransferase (COMT), which is involved in the breakdown of catecholamine neurotransmitters such as dopamine. They are used as adjuncts to treat Parkinson's disease by increasing the bioavailability of the primary drug levodopa, which is a substrate of COMT. Entacapone and tolcapone are predicted to inhibit *M.tuberculosis* enoyl-acyl carrier protein reductase (InhA), which is essential for type II fatty acid biosynthesis and the subsequent synthesis of the bacterial cell wall [16]. InhA is the target of the anti-tubercular drugs isoniazid [17] and ethionamide [18]. Similar to newly developed direct InhA inhibitors [3], [19]–[21], entacapone and tolcapone require no enzymatic activation to bind InhA. Thus they may avoid the commonly observed resistance mechanism to isoniazid and ethionamide that is exhibited by many MDR strains. Our computational predictions have been partially validated by demonstrating that the entacapone drug tablet Comtan inhibits the growth of *M.tuberculosis* at the minimal inhibition concentration (MIC_99_) of entacapone of 260.0 µM, well below the concentration leading to neuroblastoma cellular toxicity. The direct inhibition of InhA by entacapone is further confirmed by experimental enzyme kinetic assays, in which Comtan is shown to reduce InhA activity by up to 47% at the effective entacapone concentration of 80 µM. Since entacapone has an excellent safety profile with few side effects, it shows potential as a drug lead in the development of a new class of anti-tubercular therapeutics with favorable ADME/Tox properties.

## Results

### Co-factor and ligand binding site similarity between COMT and InhA

Recently, Xie and Bourne developed a sequence order independent profile-profile alignment (SOIPPA) algorithm [11], which they subsequently used to detect common binding sites among proteins unrelated in sequence and/or function. Their studies implied an evolutionary relationship between the NAD-binding Rossmann fold and several other fold classes, including the SAM-binding domain of the SAM-dependent methyltransferases, through similarities between their co-factor binding sites. It is interesting to note that both nicotinamide adenine dinucleotide (NAD) and S-adenosyl methionine (SAM) include adenine as a common molecular fragment. In fact, previous studies have shown that adenine binding pockets from proteins lacking significant homology will share common physiochemical properties [11]. These findings, plus the versatility of our method, form the basis for the present study.

**Figure 1:**
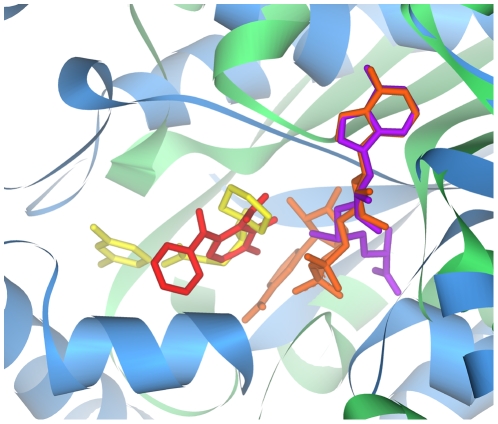
Ligand binding site similarity between COMT and InhA. COMT is show in green, its SAM co-factor is shown in purple, and its ligand is shown in red. InhA is shown in blue, its NAD co-factor is shown in orange, and its ligand is shown in yellow. Protein structures were aligned using the SOIPPA algorithm.

Entacapone and tolcapone are drugs that block the ligand binding site of COMT, a member of the SAM-dependent methyltransferase superfamily, in the presence of the SAM co-factor. They are used as adjuncts in Parkinson's disease therapy, to prevent the metabolism of levodopa to 3-*O*-methyldopa, therefore improving levodopa bioavailability and increasing its delivery to the brain [22]. The dopamine precursor levodopa has been the key drug for symptomatic treatment of Parkinson's disease for more than 30 years. Since COMT is a SAM-dependent methyltransferase, it is possible that it may possess a ligand binding pocket similar to those found in protein domains belonging to the NAD-binding Rossmann fold, as their co-factor binding sites are strikingly similar [11]. When entacapone and tolcapone were docked into 215 NAD-binding proteins from multiple species, the InhA enzyme from several different organisms, including *M.tuberculosis*, was consistently highly ranked (see Tables S1 and S2). Since InhA is the primary target of the anti-tubercular drugs isoniazid [17] and ethionamide [18], entacapone and tolcapone can potentially inhibit the InhA ligand binding site directly. Indeed, alignment of the COMT and InhA binding sites by the SOIPPA algorithm revealed similarities in the positioning of both their co-factors and ligands (Figure 1).

### 2D small molecule similarity between existing and potential InhA inhibitors

**Figure 2:**
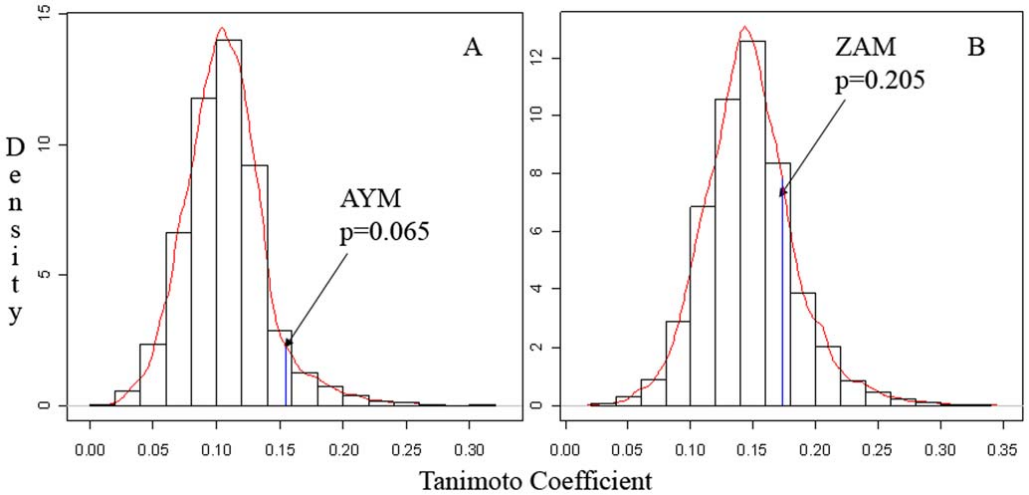
2D small molecule similarity between existing and potential InhA inhibitors. The p-value of the InhA inhibitor with the highest 2D similarity score (Tanimoto coefficient) to A) entacapone and B) tolcapone is shown against a density distribution of 15,000 background scores.

As shown in Figure 2, the existing InhA inhibitor with the greatest 2D similarity to entacapone is 3-(6-aminopyridin-3-yl)-N-methyl-N-[(1-methyl-1H-indol-2-yl)methyl]acrylamide (AYM) [23] (Tanimoto coefficient = 0.155), whereas the existing InhA inhibitor with the greatest 2D similarity to tolcapone is 3-[(acetyl-methyl-amino)-methyl]-4-amino-N-methyl-N-[(1-methyl-1H-indol-2-yl)-methyl]-benzamide (ZAM) [23] (Tanimoto coefficient = 0.173) (see Table S3). Neither of their p-values (AYM; 0.065 and ZAM; 0.205) are significant at the 0.05 level, implying that none of the investigated InhA inhibitors exhibit significant molecular similarity to either entacapone or tolcapone. Therefore, it is unlikely that ligand-based screening methods would be able to identify entacapone and tolcapone as potential InhA inhibitors.

### Docking existing and potential InhA inhibitors and drug-like molecules into InhA and COMT

Table 1 shows the predicted binding affinities of entacapone and tolcapone towards InhA. Since they fall within the range of binding affinities exhibited by the known InhA inhibitors, this not only provides a further implication of the cross-reactivity between InhA and COMT, but also suggests that entacapone and tolcapone are able to inhibit InhA directly.

**Table 1:** Docking existing and potential InhA inhibitors into InhA and COMT.

InhA inhibitor	IC_50_ to InhA (nM)	Docking score with InhA	Docking score with COMT
Pyrrolidine carboxamide s3	>100,000 [20]	−5.14+/−1.33	−6.10
468	23,120 [20]	−6.57+/−1.27	−4.42
566	10,660 [20]	−6.24+/−0.92	−3.96
Triclosan	1,000 [21]	−6.34+/−0.68	−4.05
744	970 [20]	−6.07+/−1.28	−5.47
665	890 [20]	−5.18+/−0.72	−4.20
641	390 [20]	−6.00+/−1.51	−5.92
GEQ	200 [19]	−6.29+/−1.61	−4.45
5PP	17 [21]	−5.99+/−0.48	−3.90
8PP	5 [21]	−6.51+/−0.95	−4.04
Entacapone	>80,000	−4.91+/−0.97	−4.49
Tolcapone	-	−5.85+/−0.74	−4.68

The results of the eHiTs docking studies are shown. The mean and standard deviation of the docking scores of each molecule with nine different InhAs are given, and docking scores with COMT are included as a comparison. The same docking studies using Surflex showed strong agreement (see Table S4).

Unfortunately it is difficult to identify entacapone as a lead compound using conventional virtual screening because it is only ranked at 15,892 and 9,719 by eHiTs and Surflex among 20,000 randomly selected drug-like molecules, respectively. While advanced virtual screening techniques such as the relaxed complex scheme [24], which combines a docking algorithm with molecular dynamics simulations, may improve the ranking of these potential lead compounds [25], they demand significant computational resources. Thus, SOIPPA, a ligand binding site similarity based method, provides an efficient way of identifying potential drug-like leads that have well-established pharmacokinetics and pharmacodynamics properties.

### Binding pose analysis of potential InhA inhibitors with InhA

Although the 2D similarities between entacapone and existing InhA inhibitors have been shown to be statistically insignificant, entacapone shares a similar molecular size and common functional groups with several of the InhA inhibitors (see Table 1). For example, entacapone and five of the InhA inhibitors (468, 566, 641, 665 and 774) all possess a single benzene and amide moiety. More importantly, the predicted binding poses of the benzene ring and the amide bond of entacapone are similar to those of the InhA inhibitors, with root mean square deviations (RMSDs) of as little as 2.87Å and 1.05Å, respectively (in the case of 566). Although tolcapone does not share the same amide moiety, the predicted binding pose of its benzene ring has an RMSD of only 1.01Å from that of 566, further demonstrating the potential of these drugs as InhA inhibitors.

**Figure 3:**
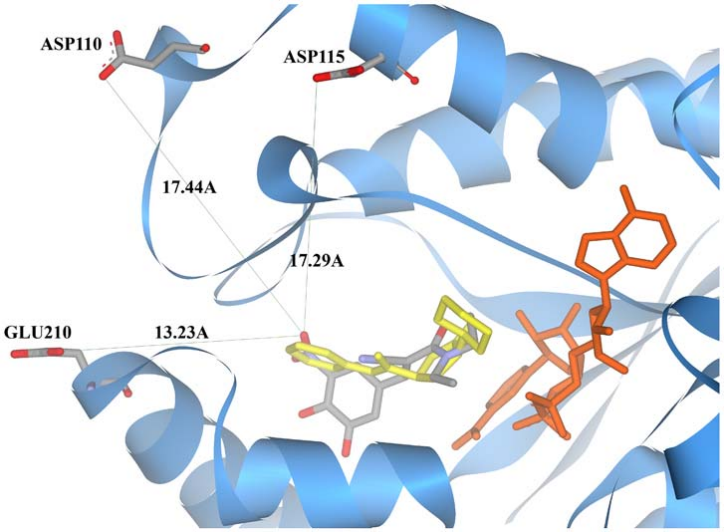
Binding pose analysis of entacapone with InhA. The eHiTs predicted binding pose of entacapone is compared with that of a native InhA ligand. The native ligand is shown in yellow and entacapone is colored by element. The NAD co-factor is shown in orange. Distances between the nitrite group of entacapone and surrounding aspartic acid and glutamic acid residues are labeled.

Previous studies have highlighted the necessity of the interaction between the catechol oxygens of COMT inhibitors with an Mg^2+^ ion in the active site [26]. From the predicted binding poses of entacapone and tolcapone docked with InhA, three potential interaction sites of Mg^2+^, including the Asp110, Asp115, and Glu210 residues of InhA, have been identified and are shown in Figure 3. The closest residue, Glu210, is positioned at a distance of 13.23Å from the nitrite group of entacapone, and at a distance of 11.54Å from the nitrite group of tolcapone (see Figure S2). Although it is possible that the conformation of the side chains may adjust to reduce this distance under *in vitro* or *in vivo* conditions, such a large distance may hinder the formation of a coordinate bond by the Mg^2+^ ion between the InhA active site and each of the two drugs. Consistent with the predicted binding pose, enzyme kinetic assays indicate that the addition of an Mg^2+^ ion has no effect on the inhibition of InhA by entacapone. This therefore provides us with opportunities to optimize entacapone and tolcapone, by reducing or removing their conjugation to the Mg^2+^ ion, so that they exhibit a weaker affinity for the original target COMT.

### Comparison of logP and logD values between existing and potential anti-tubercular drugs

The partition coefficient (logP) is the ratio of the concentrations of an unionized compound in the two phases of a mixture of octanol and water at equilibrium, whereas the distribution coefficient (logD) is the ratio of the sum of the concentrations of all forms of the compound (both ionized and unionized) in each of the two phases. Since logD is pH dependent, the pH at which it was measured is specified, as shown in Table 2. Entacapone and tolcapone were discovered to have generally higher logP and logD values than most of the existing anti-tubercular drugs investigated. According to Lipinski's rule of five [27], poor absorption or permeation is more likely for compounds with a logP value of greater than 5.0. Such compounds are considered non-drug-like and are commonly filtered out in the early stages of drug discovery. However, entacapone and tolcapone are prescribed drugs and have been clinically tested with desired pharmacokinetics profiles. Indeed, entacapone can be rapidly absorbed with a T_max_ of approximately one hour (http://www.rxlist.com/comtan-drug.htm). The high logP values of entacapone and tolcapone are therefore acceptable regardless of Lipinski's rule. Moreover, they suggest that entacapone and tolcapone are more hydrophobic than existing drugs, and would therefore pass more easily through the *M.tuberculosis* cell envelope. Although entacapone demonstrates poor solubility, dissolution enhancers such as croscarmellose sodium are used in the formulation of Comtan [28]. This example well illustrates that drug repurposing would accelerate drug discovery and development by bypassing the time consuming steps of ADME/Tox evaluation and drug formulation.

**Table 2:** Comparison of logP and logD values between existing and potential anti-tubercular drugs or pro-drugs (marked by an asterisk).

Drug		logP	logD (pH 2)	logD (pH 5)	logD (pH 7)	logD (pH 9)
COMT inhibitor	Tolcapone	3.12	3.12	2.40	0.81	0.53
	Entacapone	1.95	1.94	1.59	−0.04	−0.63
First-line anti-tubercular drug	Ethambutol	−0.31	−7.51	−6.14	−3.63	−1.31
	Isoniazid*	−0.63	−1.97	−0.64	−0.63	−0.63
	Pyrazinamide*	−1.53	−1.53	−1.53	−1.53	−1.53
	Rifampicin	2.06	−0.07	0.62	2.06	0.65
	Streptomycin	−8.30	−15.74	−15.26	−13.45	−12.05
Second-line anti-tubercular drug	Ciprofloxacin	−1.38	−2.04	−1.84	−1.38	−1.71
	Moxifloxacin	−1.88	−1.76	−1.78	−1.87	−1.87
	Aminosalicylic acid	0.81	0.54	−0.39	−2.15	−2.53

Values were calculated using ChemAxon's Calculator. Values calculated from another program ChemSilico Predict are shown in Table S5.

### Antibacterial activity of entacapone and tolcapone

Entacapone and tolcapone were evaluated for their ability to inhibit growth of *M.tuberculosis* using a 96 well microplate assay. A 99.0% reduction in growth was observed with concentrations of between 62.5 µg/ml and 125 µg/ml for each drug. The sensitivity of *M.tuberculosis* to entacapone was confirmed by quantitative growth on agar plates containing known amounts of the drug. The minimum inhibitory concentration was between 62.5 µg/ml and 125 µg/ml (Table 3), therefore confirming the result from the microplate assay. These results support the computational predictions that entacapone and tolcapone have inhibitory activity against *M.tuberculosis* and may therefore be considered as lead compounds.

**Table 3:** *In vitro* assay results for sensitivity of *M.tuberculosis* to entacapone.

Entacapone concentration (µg/ml)	Bacterial number (cfu/ml)	Percentage inhibition
None	3.5×10^6^	0.0
15.6	3.3×10^6^	6.0
31.2	2.5×10^6^	29.0
62.5	7.0×10^6^	80.0
125.0	<100.0	>99.99
250.0	<100.0	>99.99

### Enzyme kinetic assay of direct inhibition of InhA by entacapone

The ability of entacapone to directly inhibit InhA was evaluated using enzyme kinetics. Due to the strong UV absorbance of entacapone over a wide range of 300–400 nm (see Figure S4), and the poor solubility of entacapone in water, the highest concentration of Comtan that could be used in the assays was 90 µg/ml (the corresponding concentration of pure entacapone is 24.9 µg/ml), at which concentration InhA is approximately 47% inhibited. Fitting of the data in Table 4 to a dose response equation provided an IC_50_ value of 24±3 µg/ml (79±10 µM) (see Figure S3). Since Mg^2+^ is critical for entacapone and tolcapone to inhibit COMT, assays were repeated in the presence of 5 mM Mg^2+^ to explore whether or not metal ion chelation could improve the affinity of the drug for InhA. However, the inclusion of Mg^2+^ in the assay had no effect on the IC_50_ value for enzyme inhibition. The progress curve analysis for the inhibition of InhA by Comtan shows that the UV absorbance is linearly time-dependent up to one hour (see Figure S5), indicating that entacapone is not a slow-onset inhibitor.

**Table 4:** InhA kinetic assay results for entacapone inhibition.

Entacapone (µg/ml)	Percentage inhibition of InhA
2.5	10.7
6.2	23.4
12.3	35.5
24.9	47.0

## Discussion

The sharp contrast between the fatty acid biosynthetic pathway in humans (FAS-I) and that found in prokaryotes (FAS-II) has established this bacterial pathway as an attractive target for the design of new antibacterial agents [20],[29]. The FAS-II pathway in *M.tuberculosis* is involved in the production of mycolic acids, which, along with peptidoglycan and arabinogalactan, are central constituents of the mycobacterium cell wall [2],[29]. InhA catalyzes the final, rate-determining step in the fatty acid elongation cycle by converting *trans*-2-enoyl-ACP to acyl-ACP in an NADH-dependent reaction [3]. This crucial regulatory enzyme is the primary molecular target of isoniazid, which has been used as a frontline anti-tubercular agent for the past 40 years [20],[29]. As a pro-drug, the activity of isoniazid is dependent on its activation by KatG, a catalase/peroxidase enzyme. KatG oxidizes isoniazid to an acyl radical that covalently binds to NADH, and functions as a potent inhibitor of InhA. Unfortunately, it is this activation requirement that allows *M.tuberculosis* to acquire resistance to the drug. Indeed, mutations in the KatG gene account for around half of all isoniazid-resistant clinical isolates [3]. Direct InhA inhibitors that avoid this activation requirement are not susceptible to this resistance mechanism [30]. Triclosan and the diazoborines are well-known InhA inhibitors that do not require activation, but unfortunately they are not suitable for human treatment due to their respective poor solubility and toxicity [3]. However, a new class of high affinity direct InhA inhibitors, consisting of alkyl diphenyl ethers of triclosan derivatives, have been found to exhibit activity against drug-resistant strains of *M.tuberculosis* [21]. In addition to these alkyl diphenyl ethers, the arylamides [20],[31], indole-piperazines, pyrazole-based inhibitors [3], and indole-based inhibitors [23],[32], have recently been described as other classes of direct InhA inhibitors.

Using a novel computational strategy we have predicted that entacapone and tolcapone will directly inhibit InhA. Our prediction was subsequently confirmed by *in vitro* antibacterial and enzyme kinetic assays using Comtan tablets containing the active component entacapone. Thus entacapone and tolcapone are promising lead compounds against drug-resistant strains of *M.tuberculosis*. These drugs are currently in clinical use, although the association of tolcapone with hepatotoxicity has caused the drug to be placed under strict regulation in the United States [33]. Entacapone, which is not associated with the same hepatotoxic risks, is therefore more attractive as a drug lead. Interestingly, a recent study showed that when patients suffering from Parkinson's disease were treated with rifampin and isoniazid, their condition was observed to improve [34]. Since rifampin inhibits DNA-dependent RNA polymerase, this observation implies cross-reactivity between the *M.tuberculosis* InhA, the target of isoniazid, and the drug targets of Parkinson's disease, which is consistent with our predictions that InhA inhibitors can also inhibit COMT.

Recent studies have shown that triclosan can trigger the upregulation of *M.tuberculosis* detoxification mechanisms that result in its metabolism or efflux from the cell [21]. For instance, triclosan has been shown to induce the expression of an aromatic dioxygenase involved in the degradation of arenes. Since triclosan consists of a diphenyl ether structure, it is thought that the induction of this enzyme may serve to degrade, and hence detoxify, triclosan [35]. The subsequent modification of triclosan derivatives has led to high affinity alkyl diphenyl ether InhA inhibitors that upregulate neither efflux pumps nor aromatic dioxygenase [21]. Although gene transcription studies are required to determine the ability of entacapone and tolcapone to cause upregulation of the aromatic dioxygenase, it is speculated that the strong electron-withdrawing nitrite groups of these drugs may result in significantly lower reduction potentials, therefore making them less prone to oxidation by this enzyme. The narrow ranges of the MIC_99_ and the IC_50_ of Comtan support this hypothesis.

From our current experimental results, it is not possible to determine the effect of the inactive ingredients in the Comtan tablets (magnesium stearate, microcrystalline cellulose, hydroxypropyl methylcellulose, yellow iron oxide and red iron oxide, titanium dioxide, sucrose, mannitol, hydrogenated vegetable oil, polysorbate 80, glycerol 85%, croscarmellose sodium) on the growth of *M.tuberculosis*. It is unlikely that the major formulations in the Comtan tablets (magnesium stearate, microcrystalline cellulose, hydroxypropyl methylcellulose, iron oxide, titanium dioxide, sucrose) directly affect *M.tuberculosis* growth because they are the same as the active ingredients found in anti-tubercular drugs such as Rifater (isoniazid/pyrazinamide/rifampin combination tablet), Rifamate (isoniazid/rifampin tablet), and Priftin, whose active component is a rifamycin derivative (http://www.rxlist.com). Other ingredients such as polysorbate 80 and croscarmellose sodium are mainly used to enhance the dissolution and stability of entacapone [28]. It will be particularly interesting if they are active against the growth of *M.tuberculosis*, as they are commonly used food and drug additives. We have observed that a higher concentration of pure entacapone than that which is present in Comtan is required to achieve the same rate of inhibition in the InhA assay. Additional experiments such as X-ray crystallography and mass spectroscopy need to be conducted in order to investigate the precise mechanisms of action of Comtan and entacapone against *M.tuberculosis*.

Although we have demonstrated that Comtan is active against *M.tuberculosis in vitro*, further studies are required in order to transform it into an anti-tubercular therapeutic. The active component of entacapone in Comtan has an MIC_99_ for *M.tuberculosis* of approximately 80 µg/ml (262 µM), and an estimated IC_50_ value for InhA inhibition of above 80 µM. However, it exhibits very low cellular toxicity, with no effect on neuroblastoma cell lines that provide an *in vitro* model for high throughput toxicity screening [36] at concentrations of up to 500 µM [37], thus making the concentration required for *M.tuberculosis* inhibition not unreasonable for a lead compound.

The possibility that entacapone inhibits other enzymes besides InhA cannot be excluded. From our initial studies of the ligand binding site similarity network in the *M.tuberculosis* structural genome, InhA is one of the most promiscuous proteins, having a similar ligand binding site to more than 20 other enzymes [15]. This implies that an InhA inhibitor can potentially interact with multiple targets. If it is proven in future studies that entacapone can inhibit multiple targets simultaneously, the potential of entacapone as an anti-tubercular drug is even more promising, as “dirty” drugs lessen the likelihood of emergent resistance and higher clinical efficacy than exquisitely selective drugs [38],[39].

A further challenge is transforming entacapone into a nanomolar inhibitor without impacting its ADME/Tox profile. A series of direct InhA inhibitors with IC_50_'s ranging from 1 nM to greater than 100 µM are available in the RCSB Protein Data Bank (PDB) [40]. Thus it is possible to build reliable 3D QSAR models in order to guide the lead optimization process. Since entacapone has been in clinical use for many years, the accumulated knowledge of its pharmacokinetics and pharmacodynamics will be invaluable in predicting ADME/Tox properties of the compound and its derivatives.

### Conclusion

The continuing emergence of *M.tuberculosis* strains resistant to all existing, affordable drug treatments means that the development of novel, effective and inexpensive drugs is an urgent priority [3]. Our chemical systems biology approach to drug discovery revealed that Comtan, with the active component entacapone, shows potential for use as an anti-tubercular drug. Entacapone may adopt different inhibition mechanisms from the first- and second-line drugs that result in MDR and XDR *M.tuberculosis* strains. Moreover, it has an excellent safety profile with few side effects, and is commercially available. Therefore, entacapone can potentially be used as a lead compound to develop a new class of anti-tubercular drugs.

By integrating techniques from ligand binding site similarity, small molecule similarity and protein-ligand docking, our chemical systems biology approach is able to model protein-ligand interaction networks on a proteome-wide scale. The systematic use of small molecules to probe biological systems will provide us with valuable clues as to the molecular basis of cellular functions, and at the same time it will shift the conventional one-target-one-drug discovery process to a new multi-target-multi-drug paradigm.

## Methods

### Ligand binding site similarity between COMT and InhA

Previously, the SOIPPA algorithm had revealed a highly significant similarity (p-value = 2.7e-5) between the NAD binding site of the Rossmann fold and SAM binding site of the SAM methyltransferases [11]. In order to identify similar ligand binding sites adjunct to the co-factor binding site, further docking studies were carried out on the ligand binding sites of proteins that bind NAD as a co-factor. Freely available docking software eHiTs 6.2 [41] and Surflex2.1 [42] were selected due to their relatively fast speed, high accuracy and ease of automation in large-scale docking studies. It is worth noting that when preparing PDB files for any of the docking studies, NAD and SAM co-factors were added as one of residues of the protein chain.

215 non-redundant proteins with NAD co-factors were downloaded from the RCSB Protein Data Bank (PDB) [40],[43]. The ideal coordinates for entacapone and tolcapone were downloaded from DrugBank [44]. Both Surflex and eHiTs were used to dock both entacapone and tolcapone onto each of the 215 proteins, and the proteins that produced the highest docking scores were investigated further. Enoyl-acyl carrier protein reductases (InhAs) from several different organisms, including *M.tuberculosis* and *Toxoplasma gondii*, were identified as proteins to which entacapone and tolcapone showed favorable binding affinities (see Tables S1 and S2). However, the *M.tuberculosis* InhA (PDB ID: 2H7M) is the focus of this paper due to its importance as a drug target for the treatment of tuberculosis.

Unfortunately, the human COMT protein that is the drug target of entacapone and tolcapone is absent from the RCSB PDB. The only COMT structure available at the time of writing is that from the brown rat, *Rattus norvegicus*. A standard protein BLAST [45] search revealed that human and rat COMT share 81% sequence identity without insertion or deletion. Moreover, their functional site residues were found to share 100% identity (see Figure S1). Therefore, rat COMT (PDB ID: 2CL5) was used as an accurate representation of human COMT throughout this study. The SOIPPA algorithm was used to align the 2H7M structure with that of 2CL5 so that their respective NAD and SAM co-factor binding sites were aligned. The aligned proteins were then visualized using Accelrys DS Visualizer (http://www.accelrys.com/products/downloads/ds_visualizer/).

### 2D small molecule similarity between existing and potential InhA inhibitors

The RCSB PDB was queried for proteins with sequence similarity to chain A of 2H7M, using an e-value cut off of 0.0001. Between them, the resulting proteins bound a total of 22 different InhA inhibitors (including the ligand of 2H7M). OpenBabel (http://openbabel.org) was used to calculate the 2D small molecule similarity between these InhA inhibitors and both entacapone and tolcapone. In order to create background distributions for comparison, all drug-like molecules were downloaded from the ZINC database [46], and a subset of 20,000 molecules was extracted randomly. The 2D similarities of each of these molecules to both entacapone and tolcapone were calculated using OpenBabel, and density distributions of the scores were plotted using R 2.5.0 [47]. P-values corresponding to the 2D similarity scores of the 22 InhA inhibitors were subsequently calculated from both density distributions (see Table S3).

### Docking existing and potential InhA inhibitors onto InhA and COMT

Nine *M.tuberculosis* InhA structures (PDB IDs: 1P44, 1P45, 2B36, 2B37, 2H7I, 2H7L, 2H7M, 2H7N and 2H7P) were downloaded from the RCSB PDB. The ten inhibitors of these InhAs (Ligands: Pyrrolidine carboxamide s3, GEQ, TCL, 5PP, 8PS, 566, 665, 641, 744 and 468) were downloaded from the RCSB PDB as ideal coordinates. In addition, the ideal coordinates of entacapone and tolcapone were generated using CORINA (http://www.molecular-networks.com/online_demos/corina_demo.html). All twelve molecules were docked onto the nine InhA structures, as well as onto COMT using eHiTs and Surflex. The mean and standard deviation of the docking scores of each molecule with all ten of the InhAs were calculated, and the docking scores were tabulated for comparison.

### Binding pose analysis of potential InhA inhibitors with InhA

The predicted binding poses of entacapone and tolcapone with the various different *M.tuberculosis* InhAs were visualized and analyzed using Accelrys DS Visualizer. Distances between the nitrite groups of entacapone and tolcapone and the surrounding aspartic acid and glutamic acid residues of InhA were measured. RMSDs between the benzene rings and the amide bonds of entacapone and the native ligands were calculated, in addition to the RMSDs between the benzene rings of tolcapone and the native ligands.

### Comparison of logP and logD values between existing and potential anti-tubercular drugs

The ideal coordinates of entacapone, tolcapone, five first-line anti-tubercular drugs (ethambutol, isoniazid, pyrazinamide, rifampicin and streptomycin) and three second-line anti-tubercular drugs (ciprofloxacin, moxifloxacin and aminosalicylic acid) were downloaded from DrugBank [44]. Both ChemAxon's Calculator from Marvin Beans (http://www.chemaxon.com/marvin) and ChemSilico Predict (http://www.chemsilico.com) were used to calculate a) the partition coefficient (logP) and b) the distribution coefficient (logD) of all ten drug molecules.

### *In vitro* assay for sensitivity of *M.tuberculosis* to entacapone and tolcapone

In order to prepare stock solutions of each drug, one tablet of Comtan (Sandoz) containing 200 mg of entacapone, and one tablet of Tasmar (Valeant) containing 100 mg of tolcapone were each ground to a fine powder and completely dissolved in dimethylsulfoxide (DMSO). For the microplate assay, serial dilutions of each drug were made in Middlebrook 7H9 media supplemented with ADS (albumin, dextrose, and saline) and Tween 80 [48] in a volume of 100 µl. A culture of *M.tuberculosis* Erdman was grown to mid log in 7H9 plus ADS and Tween 80, and adjusted to an optical density_600_ of 0.2. 100 µl of bacteria was subsequently added to each well. The cultures were incubated for 14 days until the control wells containing only medium developed a dense layer of bacteria. Wells were visually scored for the amount of growth in comparison to the control wells. The dilution of drug that produced almost complete inhibition of growth was scored as MIC_99_. The agar plate assay was carried out as previously described [48] using Middlebrook 7H9 plates supplemented with OADC and containing known amounts of entacapone. In order to determine the MIC_99_, the number of bacteria that grew in the presence of each concentration of entacapone was compared with the number of bacteria that grew on the plate with no drug.

### InhA kinetic assays

Comtan (entacapone) tablets were ground into powder, and dissolved in DMSO. Kinetic assays using *trans*-2-dodecenoyl-Coenzyme A (DD-CoA) and wild-type InhA were performed as described previously [49]. Reactions were initiated by addition of InhA to solutions containing 250 µM NADH, 25 µM DD-CoA, 0 or 5 mM MgCl_2_ and inhibitor in 30 mM PIPES and 150 mM NaCl, pH 6.8 buffer. IC_50_ values were calculated by fitting the initial velocity data to equation 1;
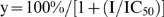
(1)where I is the inhibitor concentration and y is the percent activity. Data analysis was performed using Grafit 4.0 (Erithacus Software Ltd.). The IC_50_ curve fitting is shown in the Figure S3.

A progress curve was calculated in order to study the slow-onset mechanism of inhibition of Comtan (entacapone). InhA activity was monitored by adding the enzyme (10 nM) to assay mixtures containing 8% V/V glycerol, 0.1 mg/ml BSA, 2% V/V DMSO, 300 µM DD CoA, 250 µM NADH, 200 µM NAD^+^ and inhibitors. Reactions were monitored until the progress curve became linear, therefore indicating that the steady-state had been reached. Subsequently, a low enzyme concentration and a high substrate concentration were used to ensure that the depletion of the substrates was minimal and would not affect the reaction rate, so that the progress curve in the absence of inhibitors was linear. Progress curve data were collected for up to 1 hour (see Figure S5).

## Supporting Information

Figure S1 BLAST alignment between human and rat COMT protein sequences
(0.03 MB DOC)

Figure S2 Binding pose analysis of tolcapone with InhA
(0.48 MB DOC)

Figure S3 IC50 curve fitting for the inhibition of InhA
(0.04 MB DOC)

Figure S4 UV absorbance
(0.04 MB DOC)

Figure S5 Progressive curve
(0.06 MB DOC)

Table S1 Docking scores of entacapone with 215 NAD-binding proteins
(0.21 MB DOC)

Table S2 Docking scores of tolcapone with 215 NAD-binding proteins
(0.21 MB DOC)

Table S3 2D small molecule similarity between existing and potential InhA inhibitors
(0.06 MB DOC)

Table S4 Docking existing and potential InhA inhibitors onto InhA and COMT
(0.05 MB DOC)

Table S5 Comparison of logP and logD values between existing and potential antitubercular drugs
(0.04 MB DOC)
