# Supplementary material for: Drug Discovery Using Chemical Systems Biology: Repositioning the Safe Medicine Comtan to Treat Multi-Drug and Extensively Drug Resistant Tuberculosis
Source: PLoS Comput Biol. 2009 Jul 3;5(7):e1000423. doi: 10.1371/journal.pcbi.1000423 (PMC2699117; doi:10.1371/journal.pcbi.1000423)
Supplement: Figure S1 — BLAST alignment between human and rat COMT protein sequences (0.03 MB DOC) [file pcbi.1000423.s001.doc]

**Drug Discovery Using Chemical Systems Biology: Repositioning the safe medicine Comtan to treat multi-drug and extensively drug resistant tuberculosis**

Sarah L. Kinnings, Nina Liu, Nancy Buchmeier, Peter J. Tonge, Lei Xie, and Philip E. Bourne

**Figure S1 - BLAST alignment between human and rat COMT protein sequences**

Functional site residues are shown in red. Functional site residues are defined as those residues in 2CL5 that are a positioned at a distance of less than 6Å away from the substrate (Ligand ID: BIE).

Query: Rat COMT (PDB ID: 2CL5, chain A)

Subject: Human COMT (GeneID: 1312)

Score = 380 bits (976)

Expect = e-106

Identities = 176/217 (81%)

Positives = 202/217 (93%)

Query: 1 MGDTKEQRILRYVQQNAKPGDPQSVLEAIDTYCTQKEWAMNVGDAKGQIMDAVIREYSPS 60

MGDTKEQRIL +V Q+A+PG+ QSVLEAIDTYC QKEWAMNVGD KG+I+DAVI+E+ PS

Sbjct: 51 MGDTKEQRILNHVLQHAEPGNAQSVLEAIDTYCEQKEWAMNVGDKKGKIVDAVIQEHQPS 110

Query: 61 LVLELGAYCGYSAVRMARLLQPGARLLTMEMNPDYAAITQQMLNFAGLQDKVTILNGASQ 120

++LELGAYCGYSAVRMARLL PGARL+T+E+NPD AAITQ+M++FAG++DKVT++ GASQ

Sbjct: 111 VLLELGAYCGYSAVRMARLLSPGARLITIEINPDCAAITQRMVDFAGVKDKVTLVVGASQ 170

Query: 121 DLIPQLKKKYDVDTLDMVFLDHWKDRYLPDTLLLEKCGLLRKGTVLLADNVIVPGTPDFL 180

D+IPQLKKKYDVDTLDMVFLDHWKDRYLPDTLLLE+CGLLRKGTVLLADNVI PG PDFL

Sbjct: 171 DIIPQLKKKYDVDTLDMVFLDHWKDRYLPDTLLLEECGLLRKGTVLLADNVICPGAPDFL 230

Query: 181 AYVRGSSSFECTHYSSYLEYMKVVDGLEKAIYQGPSS 217

A+VRGSS FECTHY S+LEY +VVDGLEKAIY+GP S

Sbjct: 231 AHVRGSSCFECTHYQSFLEYREVVDGLEKAIYKGPGS 267
