# Supplementary material for: Drug Discovery Using Chemical Systems Biology: Repositioning the Safe Medicine Comtan to Treat Multi-Drug and Extensively Drug Resistant Tuberculosis
Source: PLoS Comput Biol. 2009 Jul 3;5(7):e1000423. doi: 10.1371/journal.pcbi.1000423 (PMC2699117; doi:10.1371/journal.pcbi.1000423)
Supplement: Figure S2 — Binding pose analysis of tolcapone with InhA (0.48 MB DOC) [file pcbi.1000423.s002.doc]

**Drug Discovery Using Chemical Systems Biology: Repositioning the safe medicine Comtan to treat multi-drug and extensively drug resistant tuberculosis**

Sarah L. Kinnings, Nina Liu, Nancy Buchmeier, Peter J. Tonge, Lei Xie, and Philip E. Bourne

**Figure S2 - Binding pose analysis of tolcapone with InhA**

The eHiTS predicted binding pose of tolcapone is compared with that of a native InhA ligand. The native ligand is shown in yellow and tolcapone is colored by element. The NAD co-factor is colored orange. Distances between the nitrite group of tolcapone and surrounding aspartic acid and glutamic acid residues are labeled.


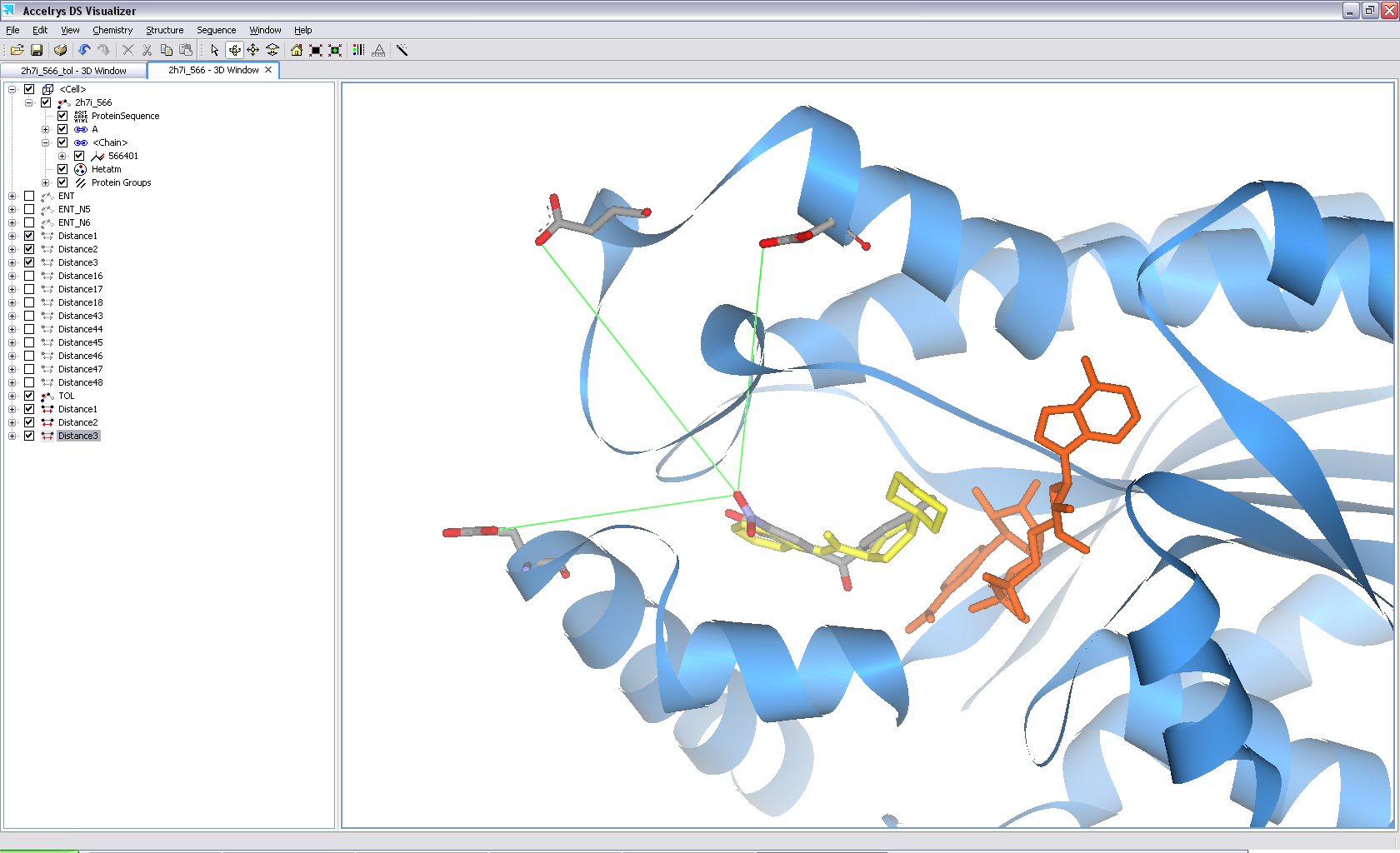


**Asp110**

**Glu210**

**Asp115**

**11.54Å**

**15.25Å**

**14.53Å**
