# Supplementary material for: Drug Discovery Using Chemical Systems Biology: Repositioning the Safe Medicine Comtan to Treat Multi-Drug and Extensively Drug Resistant Tuberculosis
Source: PLoS Comput Biol. 2009 Jul 3;5(7):e1000423. doi: 10.1371/journal.pcbi.1000423 (PMC2699117; doi:10.1371/journal.pcbi.1000423)
Supplement: Figure S3 — IC50 curve fitting for the inhibition of InhA (0.04 MB DOC) [file pcbi.1000423.s003.doc]

**Drug Discovery Using Chemical Systems Biology: Repositioning the safe medicine Comtan to treat multi-drug and extensively drug resistant tuberculosis**

Sarah L. Kinnings, Nina Liu, Nancy Buchmeier, Peter J. Tonge, Lei Xie, and Philip E. Bourne

**Figure S3 - IC50 curve fitting for the inhibition of InhA**

Data for the inhibition of InhA at various concentrations of entacapone were fit to equation 1 providing an IC_50_ value of 24 ± 3 µg/ml (79 ± 10 µM). Due to the strong UV absorbance of entacapone, the maximum concentration of drug in the assay was 25 µg/ml which is close to the IC_50_ value determined by curve fitting. Consequently, the IC_50_ value reported should be considered a lower limit.

**
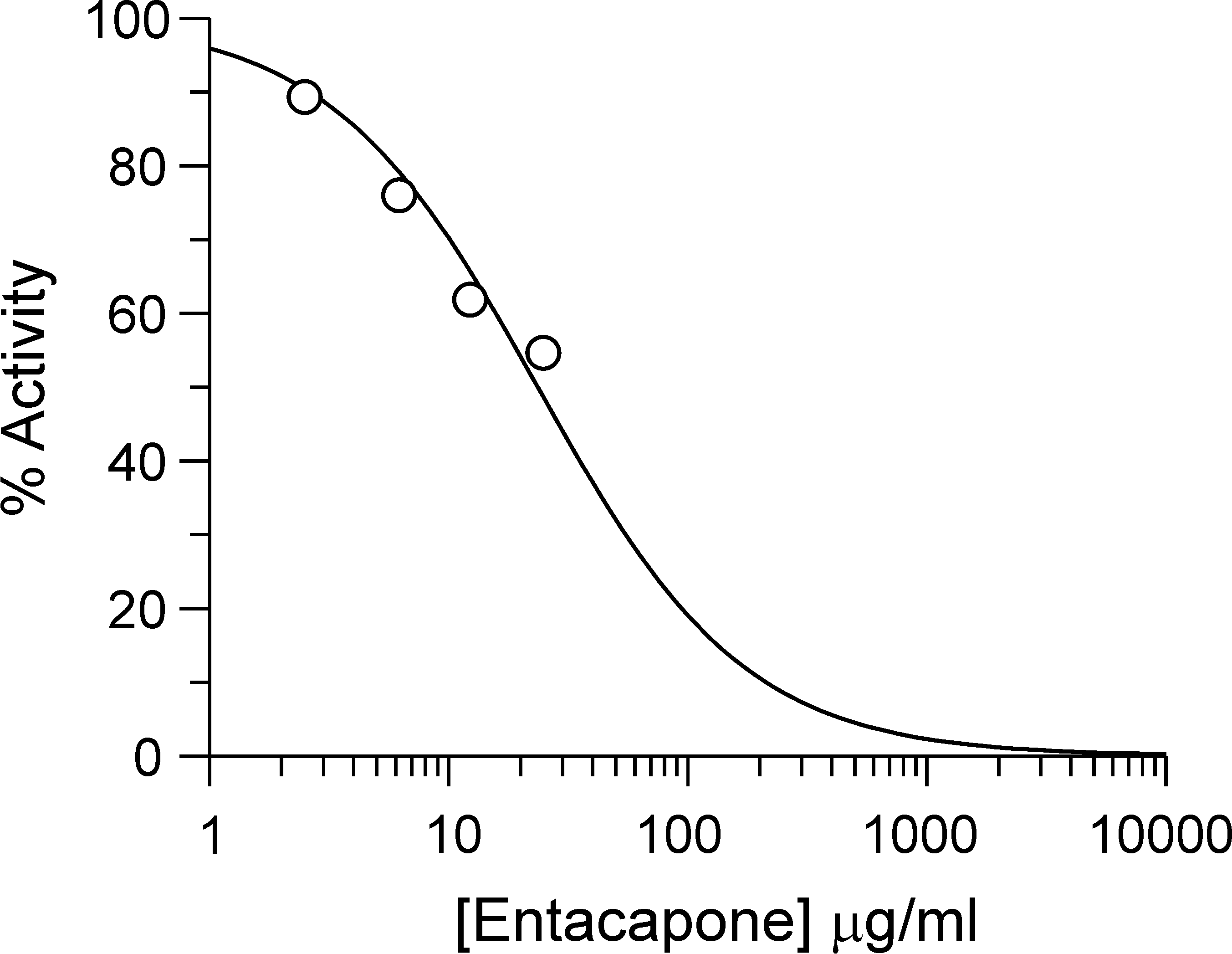
**
