# Supplementary material for: Drug Discovery Using Chemical Systems Biology: Repositioning the Safe Medicine Comtan to Treat Multi-Drug and Extensively Drug Resistant Tuberculosis
Source: PLoS Comput Biol. 2009 Jul 3;5(7):e1000423. doi: 10.1371/journal.pcbi.1000423 (PMC2699117; doi:10.1371/journal.pcbi.1000423)
Supplement: Figure S4 — UV absorbance (0.04 MB DOC) [file pcbi.1000423.s004.doc]

**Drug Discovery Using Chemical Systems Biology: Repositioning the safe medicine Comtan to treat multi-drug and extensively drug resistant tuberculosis**

Sarah L. Kinnings, Nina Liu, Nancy Buchmeier, Peter J. Tonge, Lei Xie, and Philip E. Bourne

**Figure S4.** UV absorbance of Green: NADH, Blue: Comtan 180 mg/L,

Orange: Tasmar, 89.2 mg/L, and Red: COMT inhibitor CAS #125628-97-9, 180 μM

**
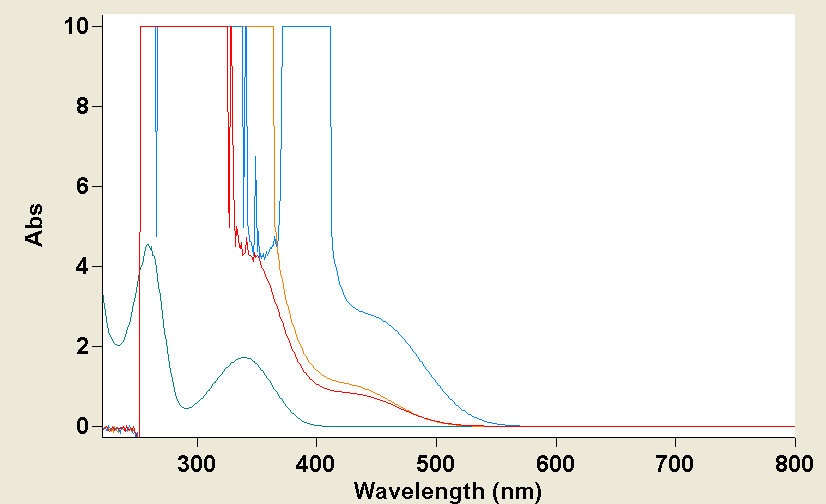
**
