# Supplementary material for: Drug Discovery Using Chemical Systems Biology: Repositioning the Safe Medicine Comtan to Treat Multi-Drug and Extensively Drug Resistant Tuberculosis
Source: PLoS Comput Biol. 2009 Jul 3;5(7):e1000423. doi: 10.1371/journal.pcbi.1000423 (PMC2699117; doi:10.1371/journal.pcbi.1000423)
Supplement: Table S1 — Docking scores of entacapone with 215 NAD-binding proteins (0.21 MB DOC) [file pcbi.1000423.s006.doc]

**Drug Discovery Using Chemical Systems Biology: Repositioning the safe medicine Comtan to treat multi-drug and extensively drug resistant tuberculosis**

Sarah L. Kinnings, Nina Liu, Nancy Buchmeier, Peter J. Tonge, Lei Xie, and Philip E. Bourne

**Table S1 - Docking scores of entacapone with 215 NAD-binding proteins**

Both eHiTS and Surflex docking scores are shown. A more negative eHiTS score indicates a stronger binding affinity, whereas a more positive Surflex score denotes a stronger binding affinity. The Surflex crash score represents the degree of inappropriate penetration into the protein by the ligand, as well as the degree of internal self-clashing experienced by the ligand. Crash scores that are close to zero are favorable. Proteins are ordered according to their eHiTS scores, and those without a score (indicated by a hyphen) failed to dock. The InhAs are shown in bold and the *M.tuberculosis* InhA is colored red.

| **PDBid** | **eHiTS score** | **Surflex score** | **(Surflex crash)** | **Protein** | **Source organism** |
| --- | --- | --- | --- | --- | --- |
| **2H7M** | **-5.89** | **4.60** | **-1.28** | **Enoyl-[acyl-carrier-protein] reductase [nadh]** | ***Mycobacterium tuberculosis*** |
| **2O2S** | **-4.70** | **3.33** | **-2.07** | **Enoyl-acyl carrier reductase** | ***Toxoplasma gondii*** |
| 1Z45 | -4.22 | 5.05 | -1.50 | Gal10 bifunctional protein | *Saccharomyces cerevisiae* |
| 1IB0 | -4.01 | 4.90 | -2.22 | Nadh-cytochrome b5 reductase | *Rattus norvegicus* |
| 1I3K | -3.96 | 3.34 | -2.31 | Udp-glucose 4-epimerase | *Homo sapiens* |
| 1HDG | -3.89 | 5.56 | -1.97 | Holo-d-glyceraldehyde-3-phosphate dehydrogenase | *Thermotoga maritima* |
| 1X1T | -3.78 | 5.42 | -2.20 | D(-)-3-hydroxybutyrate dehydrogenase | *Pseudomonas fragi* |
| 1EE9 | -3.76 | 4.12 | -3.45 | 5,10-methylenetetrahydrofolate dehydrogenase | *Saccharomyces cerevisiae* |
| 1D4F | -3.73 | 4.37 | -2.92 | S-adenosylhomocysteine hydrolase | *Rattus norvegicus* |
| 1SOW | -3.72 | -50.64 | -61.81 | L-lactate dehydrogenase | *Toxoplasma gondii* |
| 1PZH | -3.71 | 1.26 | -1.06 | Lactate dehydrogenase | *Toxoplasma gondii* |
| 1S7G | -3.63 | 3.59 | -1.68 | Nad-dependent deacetylase 2 | *Archaeoglobus fulgidus* |
| 2NPX | -3.60 | 4.50 | -4.30 | Nadh peroxidase | *Enterococcus faecalis* |
| 1J49 | -3.55 | 4.92 | -1.36 | D-lactate dehydrogenase | *Lactobacillus delbrueckii subsp. Bulgaricus* |
| 1JQ5 | -3.49 | 3.83 | -1.67 | Glycerol dehydrogenase | *Bacillus stearothermophilus* |
| **1D7O** | **-3.45** | **3.22** | **-1.20** | **Enoyl-[acyl-carrier protein] reductase (nadh) precursor** | ***Brassica napus*** |
| 2GWL | -3.43 | 2.30 | -1.35 | 65 kda virulence protein | *Salmonella typhimurium* |
| 1LLU | -3.42 | 2.80 | -1.88 | Alcohol dehydrogenase | *Pseudomonas aeruginosa* |
| 1PJC | -3.40 | 4.27 | -2.19 | Protein (l-alanine dehydrogenase) | *Phormidium lapideum* |
| 1UXT | -3.38 | 1.67 | -2.46 | Glyceraldehyde-3-phosphate dehydrogenase (nadp+) | *Thermoproteus tenax* |
| 1A5Z | -3.32 | 5.14 | -0.87 | L-lactate dehydrogenase | *Thermotoga maritima* |
| 1O04 | -3.32 | 3.76 | -1.93 | Aldehyde dehydrogenase, mitochondrial precursor | *Homo sapiens* |
| 1M9H | -3.30 | 3.29 | -2.29 | 2,5-diketo-d-gluconic acid reductase a | *Corynebacterium sp.* |
| 2DLD | -3.29 | 3.05 | -2.82 | D-lactate dehydrogenase | *Lactobacillus helveticus* |
| 1B8U | -3.29 | 3.13 | -2.70 | Protein (malate dehydrogenase) | *Aquaspirillum arcticum* |
| 1EBF | -3.27 | 5.35 | -1.10 | Homoserine dehydrogenase | *Saccharomyces cerevisiae* |
| 1MV8 | -3.23 | 2.39 | -1.34 | Gdp-mannose 6-dehydrogenase | *Pseudomonas aeruginosa* |
| 1DLI | -3.19 | 4.04 | -3.85 | Udp-glucose dehydrogenase | *Streptococcus pyogenes* |
| 1H94 | -3.15 | 2.16 | -1.12 | Glucose 6-phosphate 1-dehydrogenase | *Leuconostoc mesenteroides* |
| 1I24 | -3.14 | 2.44 | -4.54 | Sulfolipid biosynthesis protein sqd1 | *Arabidopsis thaliana* |
| 1OJS | -3.13 | 3.41 | -1.77 | Malate dehydrogenase | *Archaeoglobus fulgidus* |
| 1OC4 | -3.12 | 1.83 | -1.62 | L-lactate dehydrogenase | *Plasmodium berghei* |
| 1GYP | -3.09 | 3.81 | -2.33 | Glyceraldehyde-3-phosphate dehydrogenase | *Leishmania mexicana* |
| 1F3P | -3.09 | 4.00 | -4.07 | Ferredoxin reductase | *Pseudomonas sp.* |
| 1KAE | -3.08 | 4.06 | -2.17 | Histidinol dehydrogenase | *Escherichia coli* |
| 1VJP | -3.02 | 3.25 | -4.60 | Myo-inositol-1-phosphate synthase-related protein | *Thermotoga maritima* |
| 1GIQ | -2.99 | 0.30 | -1.32 | Iota toxin component ia | *Clostridium perfringens* |
| 1MJT | -2.98 | 3.42 | -1.52 | Nitric-oxide synthase homolog | *Staphylococcus aureus* |
| 1A4Z | -2.96 | 2.98 | -1.10 | Aldehyde dehydrogenase | *Bos taurus* |
| 1GD1 | -2.92 | 3.67 | -2.82 | Holo-d-glyceraldehyde-3-phosphate dehydrogenase | *Bacillus stearothermophilus* |
| 1EMD | -2.88 | 2.54 | -1.40 | Malate dehydrogenase | *Escherichia coli* |
| 1T2D | -2.86 | 2.38 | -3.82 | L-lactate dehydrogenase | *Plasmodium falciparum* |
| 1NFB | -2.85 | 2.21 | -5.65 | Inosine-5'-monophosphate dehydrogenase 2 | *Homo sapiens* |
| 1J5P | -2.81 | 3.31 | -2.51 | Aspartate dehydrogenase | *Thermotoga maritima* |
| 1WXH | -2.79 | 3.34 | -1.82 | Nh(3)-dependent nad(+) synthetase | *Escherichia coli* |
| 1VJT | -2.79 | 2.57 | -1.92 | Alpha-glucosidase | *Thermotoga maritima* |
| 1X31 | -2.73 | 6.85 | -4.85 | Sarcosine oxidase alpha subunit | *Corynebacterium sp.* |
| 1LLQ | -2.68 | 3.94 | -1.10 | Nad-dependent malic enzyme | *Ascaris suum* |
| 1FMC | -2.63 | 5.51 | -1.72 | 7 alpha-hydroxysteroid dehydrogenase | *Escherichia coli* |
| 1WDK | -2.63 | 4.31 | -1.64 | Fatty oxidation complex alpha subunit | *Pseudomonas fragi* |
| 1OMO | -2.60 | 3.49 | -4.28 | Alanine dehydrogenase | *Archaeoglobus fulgidus* |
| 1ML3 | -2.60 | 2.10 | -1.23 | Glyceraldehyde 3-phosphate dehydrogenase, glycosomal | *Trypanosoma cruzi* |
| 1NUU | -2.53 | 5.02 | -1.71 | Fksg76 | *Homo sapiens* |
| 2B4R | -2.52 | 1.65 | -1.45 | Glyceraldehyde-3-phosphate dehydrogenase | *Plasmodium falciparum* |
| 1BDB | -2.50 | 4.92 | -1.99 | Cis-biphenyl-2,3-dihydrodiol-2,3-dehydrogenase | *Pseudomonas sp.* |
| 1Z0Z | -2.49 | 1.83 | -1.30 | Probable inorganic polyphosphate/atp-nad kinase | *Archaeoglobus fulgidus* |
| 1ARZ | -2.49 | -0.69 | -1.92 | Dihydrodipicolinate reductase | *Escherichia coli* |
| 1OBB | -2.48 | 4.13 | -2.36 | Alpha-glucosidase | *Thermotoga maritima* |
| 1LJ8 | -2.46 | 3.85 | -1.95 | Mannitol dehydrogenase | *Pseudomonas fluorescens* |
| 1Y9E | -2.38 | 1.18 | -1.29 | Hypothetical protein yhfp | *Bacillus subtilis* |
| 1GAD | -2.38 | 5.52 | -1.15 | D-glyceraldehyde-3-phosphate dehydrogenase | *Escherichia coli* |
| 1RFM | -2.36 | 2.70 | -1.54 | L-sulfolactate dehydrogenase | *Methanococcus jannaschii* |
| 1DHS | -2.33 | 2.24 | -1.30 | Deoxyhypusine synthase | *Homo sapiens* |
| 1VBI | -2.32 | 4.53 | -5.08 | Type 2 malate/lactate dehydrogenase | *Thermus thermophilus* |
| 2BL4 | -2.32 | 2.78 | -1.93 | Lactaldehyde reductase | *Escherichia coli* |
| 1QS2 | -2.29 | 3.14 | -1.73 | Adp-ribosyltransferase | *Bacillus cereus* |
| 1HDR | -2.29 | 3.40 | -1.74 | Dihydropteridine reductase | *Homo sapiens* |
| 1PJ3 | -2.28 | 3.34 | -1.56 | Nad-dependent malic enzyme, mitochondrial | *Homo sapiens* |
| 1RKX | -2.25 | 3.54 | -1.92 | Cdp-glucose-4,6-dehydratase | *Yersinia pseudotuberculosis* |
| 1X87 | -2.22 | 2.24 | -2.24 | Urocanase protein | *Bacillus stearothermophilus* |
| 1UDC | -2.20 | 4.79 | -1.21 | Udp-galactose-4-epimerase | *Escherichia coli* |
| 1F8G | -2.19 | 5.34 | -3.02 | Nicotinamide nucleotide transhydrogenase | *Rhodospirillum rubrum* |
| 1BI9 | -2.15 | 4.98 | -1.57 | Retinal dehydrogenase type ii | *Rattus norvegicus* |
| 1DXY | -2.15 | 3.15 | -0.94 | D-2-hydroxyisocaproate dehydrogenase | *Lactobacillus casei* |
| 2DFD | -2.14 | 1.96 | -1.15 | Malate dehydrogenase | *Homo sapiens* |
| 9LDT | -2.13 | 2.16 | -1.53 | Lactate dehydrogenase | *Sus scrofa* |
| 1AD3 | -2.13 | 2.95 | -1.30 | Aldehyde dehydrogenase (class 3) | *Rattus norvegicus* |
| 1MX3 | -2.09 | 3.79 | -1.28 | C-terminal binding protein 1 | *Homo sapiens* |
| 2IZZ | -2.09 | 3.68 | -2.03 | Pyrroline-5-carboxylate reductase 1 | *Homo sapiens* |
| 2NSY | -2.09 | 1.10 | -1.29 | Protein (nad synthetase) | *Bacillus subtilis* |
| 1NBO | -2.04 | 2.99 | -3.33 | Glyceraldehyde-3-phosphate dehydrogenase a | *Spinacia oleracea* |
| 1V9L | -2.03 | 3.90 | -2.19 | Glutamate dehydrogenase | *Pyrobaculum islandicum* |
| 1O6Z | -2.02 | 2.85 | -1.31 | Malate dehydrogenase | *Haloarcula marismortui* |
| 1UXJ | -2.02 | 4.82 | -1.18 | Malate dehydrogenase | *Chloroflexus aurantiacus* |
| 1ICI | -2.01 | -0.25 | -8.82 | Transcriptional regulatory protein, sir2 family | *Archaeoglobus fulgidus* |
| 1GGA | -1.99 | 4.20 | -1.85 | D-glyceraldehyde-3-phosphate dehydrogenase | *Trypanosoma brucei brucei* |
| 1DSS | -1.99 | 2.94 | -1.70 | D-glyceraldehyde-3-phosphate-dehydrogenase | *Palinurus versicolor* |
| 1HWY | -1.95 | 4.46 | -1.67 | Glutamate dehydrogenase | *Bos taurus* |
| 1YL7 | -1.95 | 1.62 | -1.77 | Dihydrodipicolinate reductase | *Mycobacterium tuberculosis* |
| 1OG3 | -1.90 | -6.82 | -14.74 | T-cell ecto-adp-ribosyltransferase 2 | *Rattus norvegicus* |
| 1VI2 | -1.88 | 5.59 | -2.52 | Shikimate 5-dehydrogenase 2 | *Escherichia coli* |
| 1U8F | -1.87 | 1.40 | -2.13 | Glyceraldehyde-3-phosphate dehydrogenase, liver | *Homo sapiens* |
| 1BXK | -1.84 | 5.30 | -1.76 | Protein (dtdp-glucose 4,6-dehydratase) | *Escherichia coli* |
| 1OJZ | -1.84 | 2.26 | -2.16 | Adp-ribosyltransferase | *Staphylococcus aureus* |
| 1SB8 | -1.83 | 4.60 | -3.02 | Wbpp | *Pseudomonas aeruginosa* |
| 2H4F | -1.81 | 2.81 | -2.22 | Nad-dependent deacetylase | *Thermotoga maritima* |
| 1S20 | -1.80 | 4.93 | -1.04 | Hypothetical oxidoreductase yiak | *Escherichia coli* |
| 1WNB | -1.78 | 2.38 | -1.38 | Putative betaine aldehyde dehydrogenase | *Escherichia coli* |
| 1GEE | -1.76 | 2.45 | -3.51 | Glucose 1-dehydrogenase | *Bacillus megaterium* |
| 1BPW | -1.75 | 3.38 | -1.41 | Protein (aldehyde dehydrogenase) | *Gadus callarias* |
| 2A5F | -1.72 | - | - | Cholera enterotoxin, A chain | *Vibrio cholerae* |
| 1KOL | -1.69 | 2.46 | -2.12 | Formaldehyde dehydrogenase | *Pseudomonas putida* |
| 1NVM | -1.64 | 0.74 | -1.19 | 4-hydroxy-2-oxovalerate aldolase | *Pseudomonas sp.* |
| 1X0X | -1.64 | 3.73 | -1.92 | Glycerol-3-phosphate dehydrogenase [nad+], cytoplasmic | *Homo sapiens* |
| 1GEU | -1.62 | 4.23 | -2.51 | Glutathione reductase | *Escherichia coli* |
| 2GDZ | -1.60 | -1.33 | -10.07 | Nad+-dependent 15-hydroxyprostaglandin dehydrogenase | *Homo sapiens* |
| 2DT5 | -1.57 | 4.68 | -1.51 | At-rich dna-binding protein | *Thermus thermophilus* |
| 2BJK | -1.56 | 5.69 | -2.48 | 1-pyrroline-5-carboxylate dehydrogenase | *Thermus thermophilus* |
| 1PJS | -1.55 | 2.79 | -1.24 | Siroheme synthase | *Salmonella typhimurium* |
| 1LW7 | -1.53 | 6.36 | -1.44 | Transcriptional regulator nadr | *Haemophilus influenzae* |
| 1TAE | -1.52 | 3.61 | -4.73 | Dna ligase, nad-dependent | *Enterococcus faecalis v583* |
| 1GY8 | -1.51 | 4.09 | -6.51 | Udp-galactose 4-epimerase | *Trypanosoma brucei* |
| 2O2Z | -1.51 | 2.00 | -1.35 | Hypothetical protein | *Bacillus halodurans* |
| 1GRB | -1.47 | 9.67 | -5.37 | Glutathione reductase | *Homo sapiens* |
| 1VC2 | -1.41 | 4.40 | -1.75 | Glyceraldehyde 3-phosphate dehydrogenase | *Thermus thermophilus* |
| 1FDV | -1.36 | 2.63 | -4.22 | 17-beta-hydroxysteroid dehydrogenase | *Homo sapiens* |
| 2B69 | -1.33 | 4.47 | -2.59 | Udp-glucuronate decarboxylase 1 | *Homo sapiens* |
| 2G8Y | -1.32 | 3.38 | -1.65 | Malate/l-lactate dehydrogenases | *Escherichia coli* |
| 1O9J | -1.24 | 4.60 | -1.76 | Aldehyde dehydrogenase, cytosolic 1 | *Elephantulus edwardii* |
| 1DQS | -1.13 | 5.50 | -4.97 | Protein (3-dehydroquinate synthase) | *Emericella nidulans* |
| 1V59 | -1.07 | 5.62 | -3.14 | Dihydrolipoamide dehydrogenase | *Saccharomyces cerevisiae* |
| 1ISO | -0.97 | 2.65 | -1.07 | Isocitrate dehydrogenase | *Escherichia coli* |
| 2HAE | -0.96 | 0.91 | -1.93 | Malate oxidoreductase | *Thermotoga maritima* |
| 1LVL | -0.68 | 4.33 | -1.29 | Dihydrolipoamide dehydrogenase | *Pseudomonas putida* |
| 1KYQ | -0.68 | 4.52 | -1.57 | Siroheme biosynthesis protein met8 | *Saccharomyces cerevisiae* |
| 1K4M | -0.59 | 5.61 | -2.74 | Namn adenylyltransferase | *Escherichia coli* |
| 2FKN | -0.48 | 2.75 | -1.90 | Urocanate hydratase | *Bacillus subtilis* |
| 1EJ2 | -0.39 | 4.45 | -0.97 | Nicotinamide mononucleotide adenylyltransferase | *Methanobacterium thermoautotrophicum* |
| 1P1H | -0.38 | 5.52 | -2.34 | Inositol-3-phosphate synthase | *Saccharomyces cerevisiae* |
| 1UWK | -0.38 | 5.04 | -4.43 | Urocanate hydratase | *Pseudomonas putida* |
| **1UH5** | **-0.38** | **3.10** | **-3.31** | **Enoyl-acp reductase** | ***Plasmodium falciparum*** |
| 1KEP | -0.17 | 3.52 | -3.73 | Dtdp-d-glucose 4,6-dehydratase | *Streptococcus suis* |
| 1UP7 | -0.16 | 4.07 | -1.36 | 6-phospho-beta-glucosidase | *Thermotoga maritima* |
| 1T90 | 0.05 | -30.01 | -39.41 | Probable methylmalonate-semialdehyde dehydrogenase | *Bacillus subtilis* |
| 1U8X | 0.24 | 4.17 | -0.86 | Maltose-6'-phosphate glucosidase | *Bacillus subtilis* |
| 1ZEM | 0.27 | 2.91 | -2.48 | Xylitol dehydrogenase | *Gluconobacter oxydans* |
| 2D4V | 0.27 | 6.56 | -1.89 | Isocitrate dehydrogenase | *Thiobacillus thiooxidans* |
| 1KEW | 0.38 | 4.33 | -4.37 | Dtdp-d-glucose 4,6-dehydratase | *Salmonella enterica serovar typhimurium* |
| 1R6D | 0.41 | 6.10 | -2.01 | Tdp-glucose-4,6-dehydratase | *Streptomyces venezuelae* |
| 1HYH | 0.43 | 4.31 | -3.47 | L-2-hydroxyisocaproate dehydrogenase | *Lactobacillus confusus* |
| 1BXS | 0.57 | 2.99 | -1.16 | Aldehyde dehydrogenase | *Ovis aries* |
| 1LLD | 0.59 | 4.13 | -2.38 | L-lactate dehydrogenase | *Bifidobacterium longum biovar longum* |
| 1IY8 | 1.17 | 2.51 | -2.99 | Levodione reductase | *Leifsonia aquatica* |
| 1K6X | - | 5.19 | -3.90 | Nmra | *Emericella nidulans* |
| 1GR0 | - | 4.69 | -1.28 | Myo-inositol-1-phosphate synthase | *Mycobacterium tuberculosis* |
| 1RZ1 | - | 4.15 | -1.40 | Phenol 2-hydroxylase component b | *Bacillus thermoglucosidasius* |
| 1MI3 | - | 3.96 | -3.73 | Xylose reductase | *Candida tenuis* |
| 2C5A | - | 3.81 | -1.85 | Gdp-mannose-3', 5'-epimerase | *Arabidopsis thaliana* |
| 1MEW | - | 3.68 | -2.42 | Inosine-5'-monophosphate dehydrogenase | *Tritrichomonas foetus* |
| 1ZPT | **-** | 3.55 | -1.88 | 5,10-methylenetetrahydrofolate reductase | *Escherichia coli* |
| 1GEG | - | 3.54 | -3.04 | Acetoin reductase | *Klebsiella pneumoniae* |
| 2NAD | - | 3.50 | -1.44 | Nad-dependent formate dehydrogenase | *Pseudomonas sp.* |
| 1SBY | - | 3.43 | -1.71 | Alcohol dehydrogenase | *Drosophila lebanonensis* |
| 1TOX | - | 3.11 | -1.17 | Diphtheria toxin (dimeric) | *Corynephage beta* |
| 2I65 | - | 3.00 | -1.58 | Adp-ribosyl cyclase 1 | *Homo sapiens* |
| 2G5C | - | 2.88 | -2.20 | Prephenate dehydrogenase | *Aquifex aeolicus vf5* |
| 1ZMC | - | 2.75 | -1.92 | Dihydrolipoyl dehydrogenase | *Homo sapiens* |
| 1WWK | - | 2.72 | -1.77 | Phosphoglycerate dehydrogenase | *Pyrococcus horikoshii* |
| 1BMD | - | 2.72 | -1.74 | Malate dehydrogenase | *Thermus thermophilus* |
| 4MDH | - | 2.67 | -2.34 | Cytoplasmic malate dehydrogenase | *Sus scrofa* |
| 2EWM | - | 2.63 | -2.01 | (s)-1-phenylethanol dehydrogenase | *Azoarcus* |
| 1GUZ | - | 2.40 | -1.41 | Malate dehydrogenase | *Chlorobium vibrioforme, chlorobium tepi* |
| 1PL8 | - | 2.32 | -1.54 | Human sorbitol dehydrogenase | *Homo sapiens* |
| 1LSS | - | 2.31 | -1.70 | Trk system potassium uptake protein trka homolog | *Methanococcus jannaschii* |
| 2FZW | - | 2.19 | -0.88 | Alcohol dehydrogenase class iii chi chain | *Homo sapiens* |
| 2O23 | - | 2.16 | -1.27 | Hadh2 protein | *Homo sapiens* |
| 2BKJ | - | 2.16 | -1.18 | Flavin reductase | *Vibrio harveyi* |
| 2D37 | - | 2.07 | -5.34 | Hypothetical nadh-dependent fmn oxydoreductase | *Sulfolobus tokodaii str. 7* |
| 1R37 | - | 2.02 | -0.96 | Nad-dependent alcohol dehydrogenase | *Sulfolobus solfataricus* |
| 3HDH | - | 1.97 | -1.04 | Protein (l-3-hydroxyacyl coa dehydrogenase) | *Sus scrofa* |
| 1SC6 | - | 1.96 | -0.81 | D-3-phosphoglycerate dehydrogenase | *Escherichia coli* |
| 2CFC | - | 1.90 | -1.42 | 2-(r)-hydroxypropyl-com dehydrogenase | *Xanthobacter autotrophicus* |
| 1FK8 | - | 1.90 | -1.37 | 3alpha-hydroxysteroid dehydrogenase/carbonyl reductase | *Comamonas testosteroni* |
| 1F0Y | - | 1.89 | -0.87 | L-3-hydroxyacyl-coa dehydrogenase | *Homo sapiens* |
| 1U3W | - | 1.79 | -0.86 | Alcohol dehydrogenase gamma chain | *Homo sapiens* |
| 1D1T | - | 1.72 | -1.22 | Alcohol dehydrogenase class iv sigma chain | *Homo sapiens* |
| 3LDH | - | 1.70 | -1.16 | N/a | *N/a* |
| 1HSO | - | 1.66 | -1.21 | Class i alcohol dehydrogenase 1, alpha subunit | *Homo sapiens* |
| 1Z2I | - | 1.63 | -1.70 | Malate dehydrogenase | *Agrobacterium tumefaciens* |
| 1XAH | - | 1.63 | -0.76 | 3-dehydroquinate synthase | *Staphylococcus aureus* |
| 1NFF | - | 1.59 | -1.24 | Putative oxidoreductase rv2002 | *Mycobacterium tuberculosis* |
| 1VM6 | - | 1.58 | -1.26 | Dihydrodipicolinate reductase | *Thermotoga maritima* |
| 1ORR | - | 1.53 | -1.31 | Cdp-tyvelose-2-epimerase | *Salmonella typhi* |
| 1E3I | - | 1.49 | -1.03 | Alcohol dehydrogenase, class ii | *Mus musculus* |
| **1QSG** | **-** | **1.47** | **-1.03** | **Enoyl-[acyl-carrier-protein] reductase** | ***Escherichia coli*** |
| 1EVJ | - | 1.46 | -1.18 | Glucose-fructose oxidoreductase | *Zymomonas mobilis* |
| 2G76 | - | 1.44 | -0.93 | D-3-phosphoglycerate dehydrogenase | *Homo sapiens* |
| 1LDM | - | 1.41 | -2.68 | N/a | *N/a* |
| 2D1Y | - | 1.40 | -2.17 | Hypothetical protein tt0321 | *Thermus thermophilus* |
| 1EZ4 | - | 1.38 | -1.76 | Lactate dehydrogenase | *Lactobacillus pentosus* |
| 2DC1 | - | 1.37 | -1.21 | L-aspartate dehydrogenase | *Archaeoglobus fulgidus* |
| 1GV0 | - | 1.36 | -1.18 | Malate dehydrogenase | *Chlorobium tepidum* |
| 2HSD | - | 1.32 | -1.07 | 3-alpha, 20 beta-hydroxysteroid dehydrogenase | *Streptomyces exfoliatus* |
| 2GSD | - | 1.29 | -3.62 | Nad-dependent formate dehydrogenase | *Moraxella sp.* |
| 1CDO | - | 1.27 | -1.24 | Alcohol dehydrogenase | *Gadus callarias* |
| 1E6W | - | 1.26 | -1.45 | Short chain 3-hydroxyacyl-coa dehydrogenase | *Rattus norvegicus* |
| 2CZC | - | 1.15 | -1.10 | Glyceraldehyde-3-phosphate dehydrogenase | *Pyrococcus horikoshii* |
| 3GPD | - | 1.10 | -1.11 | D-glyceraldehyde-3-phosphate dehydrogenase | *Homo sapiens* |
| 2AG5 | **-** | 1.08 | -0.95 | Dehydrogenase/reductase (sdr family) member 6 | *Homo sapiens* |
| 1LDN | - | 1.05 | -0.99 | L-lactate dehydrogenase | *Bacillus stearothermophilus* |
| 1HEX | - | 0.94 | -1.06 | 3-isopropylmalate dehydrogenase | *Thermus thermophilus* |
| 1ZBQ | - | 0.89 | -1.60 | 17-beta-hydroxysteroid dehydrogenase 4 | *Homo sapiens* |
| 1U3U | - | 0.55 | -0.86 | Alcohol dehydrogenase beta chain | *Homo sapiens* |
| 2I9P | - | 0.52 | -1.06 | 3-hydroxyisobutyrate dehydrogenase | *Homo sapiens* |
| 1T2F | - | 0.46 | -0.75 | L-lactate dehydrogenase b chain | *Homo sapiens* |
| 1QAY | - | 0.39 | -1.06 | Protein (3-hydroxy-3-methylglutaryl-coenzyme a reductase) | *Pseudomonas mevalonii* |
| 1KQN | - | 0.14 | -1.04 | Nicotinamide mononucleotide adenylyl transferase | *Homo sapiens* |
| 1X14 | - | 0.11 | -1.28 | Nad(p) transhydrogenase subunit alpha | *Escherichia coli* |
| 1Y3I | - | -0.07 | -0.71 | Inorganic polyphosphate/atp-nad kinase | *Mycobacterium tuberculosis* |
| 2GAG | - | -0.24 | -0.92 | Heterotetrameric sarcosine oxidase alpha-subunit | *Xanthomonas maltophilia* |
| 2A9K | **-** | -3.76 | -0.80 | Ras-related protein ral-a | *Homo sapiens* |
| 1X7D | - | -7.20 | -17.08 | Ornithine cyclodeaminase | *Pseudomonas putida* |
| 1U1I | - | -11.71 | -16.49 | Myo-inositol-1-phosphate synthase | *Archaeoglobus fulgidus dsm 4304* |
| 1VKO | - | -22.98 | -33.25 | Inositol-3-phosphate synthase | *Caenorhabditis elegans* |
| 1OWB | - | - | - | Citrate synthase | *Escherichia coli* |
