# Supplementary material for: Drug Discovery Using Chemical Systems Biology: Repositioning the Safe Medicine Comtan to Treat Multi-Drug and Extensively Drug Resistant Tuberculosis
Source: PLoS Comput Biol. 2009 Jul 3;5(7):e1000423. doi: 10.1371/journal.pcbi.1000423 (PMC2699117; doi:10.1371/journal.pcbi.1000423)
Supplement: Table S2 — Docking scores of tolcapone with 215 NAD-binding proteins (0.21 MB DOC) [file pcbi.1000423.s007.doc]

**Drug Discovery Using Chemical Systems Biology: Repositioning the safe medicine Comtan to treat multi-drug and extensively drug resistant tuberculosis**

Sarah L. Kinnings, Nina Liu, Nancy Buchmeier, Peter J. Tonge, Lei Xie, and Philip E. Bourne

**Table S2 - Docking scores of tolcapone with 215 NAD-binding proteins**

Both eHiTS and Surflex docking scores are shown. A more negative eHiTS score indicates a stronger binding affinity, whereas a more positive Surflex score denotes a stronger binding affinity. The Surflex crash score represents the degree of inappropriate penetration into the protein by the ligand, as well as the degree of internal self-clashing that the ligand is experiencing. Crash scores that are close to zero are favorable. Proteins are ordered according to their eHiTS scores, and those without a score (indicated by a hyphen) failed to dock. The InhAs are shown in bold and the *M.tuberculosis* InhA is colored red.

| **PDBid** | **eHiTS score** | **Surflex score** | **(Surflex crash)** | **Protein** | **Source organism** |
| --- | --- | --- | --- | --- | --- |
| 1EJ2 | -6.31 | 4.94 | -0.53 | Nicotinamide mononucleotide adenylyltransferase | *Methanobacterium thermoautotrophicum* |
| 1EE9 | -5.21 | 4.67 | -1.91 | 5,10-methylenetetrahydrofolate dehydrogenase | *Saccharomyces cerevisiae* |
| **2O2S** | **-5.01** | **4.75** | **-2.42** | **Enoyl-acyl carrier reductase** | ***Toxoplasma gondii*** |
| **2H7M** | **-4.81** | **4.16** | **-1.64** | **Enoyl-[acyl-carrier-protein] reductase [nadh]** | ***Mycobacterium tuberculosis*** |
| 1FDV | -4.31 | 4.65 | -2.16 | 17-beta-hydroxysteroid dehydrogenase | *Homo sapiens* |
| **1D7O** | **-4.29** | **4.43** | **-1.65** | **Enoyl-[acyl-carrier protein] reductase (nadh) precursor** | ***Brassica napus*** |
| 2G8Y | -4.15 | 3.38 | -0.72 | Malate/l-lactate dehydrogenases | *Escherichia coli* |
| 1PJC | -4.12 | 3.69 | -0.98 | Protein (l-alanine dehydrogenase) | *Phormidium lapideum* |
| 1OJS | -4.03 | 4.46 | -1.27 | Malate dehydrogenase | *Archaeoglobus fulgidus* |
| 1S7G | -3.93 | 3.45 | -1.35 | Nad-dependent deacetylase 2 | *Archaeoglobus fulgidus* |
| 1T2D | -3.76 | 3.34 | -0.76 | L-lactate dehydrogenase | *Plasmodium falciparum* |
| 1F8G | -3.75 | 6.26 | -0.85 | Nicotinamide nucleotide transhydrogenase | *Rhodospirillum rubrum* |
| 1BDB | -3.75 | 5.17 | -1.56 | Cis-biphenyl-2,3-dihydrodiol-2,3-dehydrogenase | *Pseudomonas sp.* |
| 1VC2 | -3.74 | 3.71 | -1.15 | Glyceraldehyde 3-phosphate dehydrogenase | *Thermus thermophilus* |
| 1B8U | -3.66 | 6.33 | -1.25 | Protein (malate dehydrogenase) | *Aquaspirillum arcticum* |
| 1EBF | -3.60 | 5.61 | -0.80 | Homoserine dehydrogenase | *Saccharomyces cerevisiae* |
| 1NBO | -3.58 | 4.80 | -1.17 | Glyceraldehyde-3-phosphate dehydrogenase a | *Spinacia oleracea* |
| 1D4F | -3.51 | 3.74 | -1.57 | S-adenosylhomocysteine hydrolase | *Rattus norvegicus* |
| 1S20 | -3.50 | 5.08 | -1.24 | Hypothetical oxidoreductase yiak | *Escherichia coli* |
| 2GWL | -3.47 | 4.06 | -0.75 | 65 kda virulence protein | *Salmonella typhimurium* |
| 1GGA | -3.44 | 2.66 | -1.01 | D-glyceraldehyde-3-phosphate dehydrogenase | *Trypanosoma brucei brucei* |
| 1HDG | -3.43 | 3.75 | -0.80 | Holo-d-glyceraldehyde-3-phosphate dehydrogenase | *Thermotoga maritima* |
| 1PZH | -3.43 | 2.24 | -0.44 | Lactate dehydrogenase | *Toxoplasma gondii* |
| 1DSS | -3.34 | 3.02 | -0.63 | D-glyceraldehyde-3-phosphate-dehydrogenase | *Palinurus versicolor* |
| 1F3P | -3.25 | 5.65 | -2.67 | Ferredoxin reductase | *Pseudomonas sp.* |
| 1J49 | -3.24 | 5.15 | -1.76 | D-lactate dehydrogenase | *Lactobacillus delbrueckii subsp. Bulgaricus* |
| 1I3K | -3.23 | 5.09 | -1.09 | Udp-glucose 4-epimerase | *Homo sapiens* |
| 1GD1 | -3.21 | 4.36 | -2.17 | Holo-d-glyceraldehyde-3-phosphate dehydrogenase | *Bacillus stearothermophilus* |
| 1KAE | -3.18 | 2.58 | -0.64 | Histidinol dehydrogenase | *Escherichia coli* |
| 1BPW | -3.18 | 3.30 | -1.56 | Protein (aldehyde dehydrogenase) | *Gadus callarias* |
| 1O6Z | -3.17 | 3.91 | -1.44 | Malate dehydrogenase | *Haloarcula marismortui* |
| 1NFB | -3.11 | 5.34 | -1.90 | Inosine-5'-monophosphate dehydrogenase 2 | *Homo sapiens* |
| 1LJ8 | -3.09 | 4.83 | -2.05 | Mannitol dehydrogenase | *Pseudomonas fluorescens* |
| 1M9H | -3.09 | 4.67 | -0.63 | 2,5-diketo-d-gluconic acid reductase a | *Corynebacterium sp.* |
| 1QS2 | -3.08 | 2.27 | -0.49 | Adp-ribosyltransferase | *Bacillus cereus* |
| 1VI2 | -3.06 | 5.08 | -2.32 | Shikimate 5-dehydrogenase 2 | *Escherichia coli* |
| 2DLD | -3.04 | 2.69 | -1.56 | D-lactate dehydrogenase | *Lactobacillus helveticus* |
| 1VJP | -3.02 | 5.38 | -1.35 | Myo-inositol-1-phosphate synthase-related protein | *Thermotoga maritima* |
| 2B4R | -3.01 | 2.08 | -0.61 | Glyceraldehyde-3-phosphate dehydrogenase | *Plasmodium falciparum* |
| 2NPX | -3.01 | 5.07 | -2.43 | Nadh peroxidase | *Enterococcus faecalis* |
| 1GRB | -2.98 | 11.48 | -4.27 | Glutathione reductase | *Homo sapiens* |
| 1DQS | -2.97 | 6.64 | -3.42 | Protein (3-dehydroquinate synthase) | *Emericella nidulans* |
| 1WXH | -2.97 | 4.22 | -0.88 | Nh(3)-dependent nad(+) synthetase | *Escherichia coli* |
| 1GAD | -2.96 | 4.70 | -1.43 | D-glyceraldehyde-3-phosphate dehydrogenase | *Escherichia coli* |
| 1OMO | -2.94 | 5.35 | -1.60 | Alanine dehydrogenase | *Archaeoglobus fulgidus* |
| 1UXT | -2.90 | 2.97 | -1.56 | Glyceraldehyde-3-phosphate dehydrogenase (nadp+) | *Thermoproteus tenax* |
| 1EMD | -2.87 | 2.04 | -1.27 | Malate dehydrogenase | *Escherichia coli* |
| 1U8F | -2.86 | 1.81 | -0.38 | Glyceraldehyde-3-phosphate dehydrogenase, liver | *Homo sapiens* |
| 1IB0 | -2.84 | 5.45 | -0.52 | Nadh-cytochrome b5 reductase | *Rattus norvegicus* |
| 1RKX | -2.81 | 5.20 | -0.92 | Cdp-glucose-4,6-dehydratase | *Yersinia pseudotuberculosis* |
| 1UXJ | -2.81 | 3.82 | -0.43 | Malate dehydrogenase | *Chloroflexus aurantiacus* |
| 1GYP | -2.80 | 3.31 | -0.53 | Glyceraldehyde-3-phosphate dehydrogenase | *Leishmania mexicana* |
| 2BL4 | -2.76 | 3.27 | -0.58 | Lactaldehyde reductase | *Escherichia coli* |
| 1HYH | -2.76 | 4.25 | -1.54 | L-2-hydroxyisocaproate dehydrogenase | *Lactobacillus confusus* |
| 1NUU | -2.75 | 3.92 | -1.34 | Fksg76 | *Homo sapiens* |
| 1RFM | -2.66 | 3.55 | -1.16 | L-sulfolactate dehydrogenase | *Methanococcus jannaschii* |
| 1DLI | -2.66 | 3.69 | -3.19 | Udp-glucose dehydrogenase | *Streptococcus pyogenes* |
| 2GDZ | -2.66 | 4.70 | -3.03 | Nad+-dependent 15-hydroxyprostaglandin dehydrogenase | *Homo sapiens* |
| 1OC4 | -2.65 | 2.41 | -0.61 | L-lactate dehydrogenase | *Plasmodium berghei* |
| 1A5Z | -2.64 | 3.92 | -1.02 | L-lactate dehydrogenase | *Thermotoga maritima* |
| 1OG3 | -2.64 | -5.41 | -14.28 | T-cell ecto-adp-ribosyltransferase 2 | *Rattus norvegicus* |
| 1SOW | -2.59 | -24.31 | -33.37 | L-lactate dehydrogenase | *Toxoplasma gondii* |
| 1DHS | -2.58 | 1.51 | -0.76 | Deoxyhypusine synthase | *Homo sapiens* |
| 1AD3 | -2.55 | 4.29 | -1.87 | Aldehyde dehydrogenase (class 3) | *Rattus norvegicus* |
| 1J5P | -2.55 | 4.00 | -0.44 | Aspartate dehydrogenase | *Thermotoga maritima* |
| 1MJT | -2.54 | 3.58 | -1.97 | Nitric-oxide synthase homolog | *Staphylococcus aureus* |
| 1Y9E | -2.53 | 2.93 | -1.35 | Hypothetical protein yhfp | *Bacillus subtilis* |
| 1MV8 | -2.52 | 2.27 | -0.63 | Gdp-mannose 6-dehydrogenase | *Pseudomonas aeruginosa* |
| 1X1T | -2.52 | 5.45 | -1.08 | D(-)-3-hydroxybutyrate dehydrogenase | *Pseudomonas fragi* |
| 1ARZ | -2.52 | 0.55 | -0.56 | Dihydrodipicolinate reductase | *Escherichia coli* |
| 1NVM | -2.49 | 1.75 | -0.67 | 4-hydroxy-2-oxovalerate aldolase | *Pseudomonas sp.* |
| 1FMC | -2.49 | 5.49 | -1.15 | 7 alpha-hydroxysteroid dehydrogenase | *Escherichia coli* |
| 1LLQ | -2.48 | 4.63 | -1.23 | Nad-dependent malic enzyme | *Ascaris suum* |
| 1GEU | -2.47 | 4.28 | -1.53 | Glutathione reductase | *Escherichia coli* |
| 1DXY | -2.42 | 4.30 | -0.50 | D-2-hydroxyisocaproate dehydrogenase | *Lactobacillus casei* |
| 2BJK | -2.38 | 4.08 | -1.65 | 1-pyrroline-5-carboxylate dehydrogenase | *Thermus thermophilus* |
| 2A5F | -2.34 | - | - | Cholera enterotoxin, A chain | *Vibrio cholerae* |
| 1T90 | -2.34 | -14.08 | -24.70 | Probable methylmalonate-semialdehyde dehydrogenase | *Bacillus subtilis* |
| 9LDT | -2.33 | 1.18 | -0.48 | Lactate dehydrogenase | *Sus scrofa* |
| 1JQ5 | -2.32 | 4.37 | -0.76 | Glycerol dehydrogenase | *Bacillus stearothermophilus* |
| 2DFD | -2.31 | 3.68 | -0.89 | Malate dehydrogenase | *Homo sapiens* |
| 1Z0Z | -2.31 | 3.31 | -0.97 | Probable inorganic polyphosphate/atp-nad kinase | *Archaeoglobus fulgidus* |
| 1HWY | -2.30 | 3.20 | -0.70 | Glutamate dehydrogenase | *Bos taurus* |
| 1HDR | -2.29 | 3.84 | -1.27 | Dihydropteridine reductase | *Homo sapiens* |
| 1UDC | -2.29 | 5.56 | -2.93 | Udp-galactose-4-epimerase | *Escherichia coli* |
| 1ML3 | -2.27 | 4.44 | -0.71 | Glyceraldehyde 3-phosphate dehydrogenase, glycosomal | *Trypanosoma cruzi* |
| 1OJZ | -2.22 | 3.00 | -0.81 | Adp-ribosyltransferase | *Staphylococcus aureus* |
| 1ISO | -2.21 | 3.31 | -2.67 | Isocitrate dehydrogenase | *Escherichia coli* |
| 2DT5 | -2.21 | 3.21 | -1.68 | At-rich dna-binding protein | *Thermus thermophilus* |
| 1VJT | -2.19 | 3.51 | -0.67 | Alpha-glucosidase | *Thermotoga maritima* |
| 1WDK | -2.17 | 3.97 | -0.95 | Fatty oxidation complex alpha subunit | *Pseudomonas fragi* |
| 1PJ3 | -2.16 | 4.56 | -1.66 | Nad-dependent malic enzyme, mitochondrial | *Homo sapiens* |
| 1OBB | -2.15 | 4.48 | -0.78 | Alpha-glucosidase | *Thermotoga maritima* |
| 1Z45 | -2.06 | 4.86 | -0.92 | Gal10 bifunctional protein | *Saccharomyces cerevisiae* |
| 1R6D | -2.04 | 4.91 | -2.23 | Tdp-glucose-4,6-dehydratase | *Streptomyces venezuelae* |
| 1YL7 | -2.00 | 2.82 | -1.15 | Dihydrodipicolinate reductase | *Mycobacterium tuberculosis* |
| 1X31 | -1.97 | 5.04 | -4.86 | Sarcosine oxidase alpha subunit | *Corynebacterium sp.* |
| 1ICI | -1.95 | 1.69 | -7.88 | Transcriptional regulatory protein, sir2 family | *Archaeoglobus fulgidus* |
| 1I24 | -1.94 | 5.32 | -1.76 | Sulfolipid biosynthesis protein sqd1 | *Arabidopsis thaliana* |
| 1IY8 | -1.86 | 3.63 | -2.92 | Levodione reductase | *Leifsonia aquatica* |
| 1LW7 | -1.85 | 5.33 | -0.92 | Transcriptional regulator nadr | *Haemophilus influenzae* |
| 2O2Z | -1.80 | 4.02 | -0.80 | Hypothetical protein | *Bacillus halodurans* |
| 1O9J | -1.76 | 3.34 | -1.82 | Aldehyde dehydrogenase, cytosolic 1 | *Elephantulus edwardii* |
| 1V9L | -1.75 | 4.13 | -0.86 | Glutamate dehydrogenase | *Pyrobaculum islandicum* |
| 1BI9 | -1.75 | 7.73 | -1.69 | Retinal dehydrogenase type ii | *Rattus norvegicus* |
| 1KOL | -1.73 | 2.86 | -1.59 | Formaldehyde dehydrogenase | *Pseudomonas putida* |
| 1PJS | -1.66 | 2.82 | -0.49 | Siroheme synthase | *Salmonella typhimurium* |
| 1P1H | -1.65 | 4.72 | -2.51 | Inositol-3-phosphate synthase | *Saccharomyces cerevisiae* |
| 1BXK | -1.64 | 3.36 | -2.03 | Protein (dtdp-glucose 4,6-dehydratase) | *Escherichia coli* |
| 2H4F | -1.62 | 4.22 | -1.70 | Nad-dependent deacetylase | *Thermotoga maritima* |
| 2IZZ | -1.62 | 2.55 | -0.94 | Pyrroline-5-carboxylate reductase 1 | *Homo sapiens* |
| 1A4Z | -1.61 | 3.20 | -0.46 | Aldehyde dehydrogenase | *Bos taurus* |
| 1WNB | -1.55 | 4.00 | -1.35 | Putative betaine aldehyde dehydrogenase | *Escherichia coli* |
| 1GEE | -1.54 | 3.83 | -2.39 | Glucose 1-dehydrogenase | *Bacillus megaterium* |
| 1VBI | -1.52 | 6.24 | -3.28 | Type 2 malate/lactate dehydrogenase | *Thermus thermophilus* |
| 1H94 | -1.51 | 4.68 | -1.08 | Glucose 6-phosphate 1-dehydrogenase | *Leuconostoc mesenteroides* |
| 2HAE | -1.49 | 2.39 | -0.58 | Malate oxidoreductase | *Thermotoga maritima* |
| 1SB8 | -1.47 | 5.74 | -1.92 | Wbpp | *Pseudomonas aeruginosa* |
| 2NSY | -1.46 | 2.27 | -1.38 | Protein (nad synthetase) | *Bacillus subtilis* |
| 1MX3 | -1.46 | 3.41 | -1.57 | C-terminal binding protein 1 | *Homo sapiens* |
| 1GIQ | -1.42 | 3.78 | -0.76 | Iota toxin component ia | *Clostridium perfringens* |
| 1GY8 | -1.40 | 4.89 | -0.89 | Udp-galactose 4-epimerase | *Trypanosoma brucei* |
| 1TAE | -1.39 | 3.88 | -1.82 | Dna ligase, nad-dependent | *Enterococcus faecalis v583* |
| 2FKN | -1.32 | 3.16 | -0.58 | Urocanate hydratase | *Bacillus subtilis* |
| 1KYQ | -1.30 | 3.28 | -2.62 | Siroheme biosynthesis protein met8 | *Saccharomyces cerevisiae* |
| 2B69 | -1.30 | 4.97 | -1.48 | Udp-glucuronate decarboxylase 1 | *Homo sapiens* |
| 1X0X | -1.27 | 4.97 | -0.81 | Glycerol-3-phosphate dehydrogenase [nad+], cytoplasmic | *Homo sapiens* |
| 1KEP | -1.22 | 5.12 | -2.66 | Dtdp-d-glucose 4,6-dehydratase | *Streptococcus suis* |
| 1ZEM | -1.16 | 4.55 | -0.93 | Xylitol dehydrogenase | *Gluconobacter oxydans* |
| 1X87 | -0.96 | 2.70 | -0.55 | Urocanase protein | *Bacillus stearothermophilus* |
| 1RZ1 | -0.82 | 4.54 | -0.60 | Phenol 2-hydroxylase component b | *Bacillus thermoglucosidasius* |
| 1LLD | -0.77 | 4.48 | -1.50 | L-lactate dehydrogenase | *Bifidobacterium longum biovar longum* |
| 1KEW | -0.76 | 6.73 | -1.41 | Dtdp-d-glucose 4,6-dehydratase | *Salmonella enterica serovar typhimurium* |
| 1U8X | -0.65 | 6.02 | -1.02 | Maltose-6'-phosphate glucosidase | *Bacillus subtilis* |
| 1V59 | -0.61 | 4.77 | -1.32 | Dihydrolipoamide dehydrogenase | *Saccharomyces cerevisiae* |
| 2D4V | -0.53 | 6.23 | -1.63 | Isocitrate dehydrogenase | *Thiobacillus thiooxidans* |
| 2C5A | -0.50 | 5.39 | -0.58 | Gdp-mannose-3', 5'-epimerase | *Arabidopsis thaliana* |
| 1K4M | -0.45 | 6.32 | -1.09 | Namn adenylyltransferase | *Escherichia coli* |
| 1UP7 | -0.40 | 4.63 | -1.44 | 6-phospho-beta-glucosidase | *Thermotoga maritima* |
| 1UWK | -0.38 | 5.04 | -1.99 | Urocanate hydratase | *Pseudomonas putida* |
| 1LLU | -0.26 | 2.84 | -1.82 | Alcohol dehydrogenase | *Pseudomonas aeruginosa* |
| 1O04 | -0.12 | 4.31 | -2.18 | Aldehyde dehydrogenase, mitochondrial precursor | *Homo sapiens* |
| **1UH5** | **0.09** | **4.19** | **-1.34** | **Enoyl-acp reductase** | ***Plasmodium falciparum*** |
| 1ZMC | - | 5.99 | -2.88 | Dihydrolipoyl dehydrogenase | *Homo sapiens* |
| 1MI3 | - | 5.79 | -0.58 | Xylose reductase | *Candida tenuis* |
| 1LVL | - | 5.63 | -1.21 | Dihydrolipoamide dehydrogenase | *Pseudomonas putida* |
| 1K6X | - | 4.99 | -0.88 | Nmra | *Emericella nidulans* |
| 1BXS | - | 4.81 | -2.56 | Aldehyde dehydrogenase | *Ovis aries* |
| 1GR0 | - | 4.79 | -1.36 | Myo-inositol-1-phosphate synthase | *Mycobacterium tuberculosis* |
| 1ZPT | - | 4.23 | -2.00 | 5,10-methylenetetrahydrofolate reductase | *Escherichia coli* |
| 2GSD | - | 4.19 | -1.11 | Nad-dependent formate dehydrogenase | *Moraxella sp.* |
| 2D37 | - | 4.18 | -2.86 | Hypothetical nadh-dependent fmn oxydoreductase | *Sulfolobus tokodaii str. 7* |
| 2NAD | - | 4.15 | -0.95 | Nad-dependent formate dehydrogenase | *Pseudomonas sp.* |
| 1EVJ | - | 4.14 | -0.64 | Glucose-fructose oxidoreductase | *Zymomonas mobilis* |
| 1SBY | - | 4.01 | -1.68 | Alcohol dehydrogenase | *Drosophila lebanonensis* |
| 1LSS | - | 3.79 | -0.45 | Trk system potassium uptake protein trka homolog | *Methanococcus jannaschii* |
| 1GEG | - | 3.78 | -1.10 | Acetoin reductase | *Klebsiella pneumoniae* |
| 1R37 | - | 3.66 | -0.59 | Nad-dependent alcohol dehydrogenase | *Sulfolobus solfataricus* |
| 1E3I | - | 3.59 | -0.84 | Alcohol dehydrogenase, class ii | *Mus musculus* |
| 1MEW | - | 3.47 | -0.40 | Inosine-5'-monophosphate dehydrogenase | *Tritrichomonas foetus* |
| 1TOX | - | 3.46 | -0.86 | Diphtheria toxin (dimeric) | *Corynephage beta* |
| 2O23 | - | 3.37 | -0.45 | Hadh2 protein | *Homo sapiens* |
| 2AG5 | - | 3.18 | -0.56 | Dehydrogenase/reductase (sdr family) member 6 | *Homo sapiens* |
| 2I65 | - | 3.17 | -0.65 | Adp-ribosyl cyclase 1 | *Homo sapiens* |
| 1CDO | - | 3.10 | -0.67 | Alcohol dehydrogenase | *Gadus callarias* |
| 2CFC | - | 3.08 | -0.48 | 2-(r)-hydroxypropyl-com dehydrogenase | *Xanthobacter autotrophicus* |
| 1VM6 | - | 3.04 | -0.59 | Dihydrodipicolinate reductase | *Thermotoga maritima* |
| 2FZW | - | 2.97 | -0.53 | Alcohol dehydrogenase class iii chi chain | *Homo sapiens* |
| 1WWK | - | 2.89 | -0.81 | Phosphoglycerate dehydrogenase | *Pyrococcus horikoshii* |
| 1NFF | - | 2.89 | -0.56 | Putative oxidoreductase rv2002 | *Mycobacterium tuberculosis* |
| 3HDH | - | 2.84 | -0.46 | Protein (l-3-hydroxyacyl coa dehydrogenase) | *Sus scrofa* |
| 1BMD | - | 2.82 | -0.51 | Malate dehydrogenase | *Thermus thermophilus* |
| 1E6W | - | 2.79 | -0.49 | Short chain 3-hydroxyacyl-coa dehydrogenase | *Rattus norvegicus* |
| 4MDH | - | 2.76 | -0.44 | Cytoplasmic malate dehydrogenase | *Sus scrofa* |
| 1F0Y | - | 2.76 | -0.74 | L-3-hydroxyacyl-coa dehydrogenase | *Homo sapiens* |
| 1T2F | - | 2.76 | -0.57 | L-lactate dehydrogenase b chain | *Homo sapiens* |
| **1QSG** | **-** | **2.73** | **-0.70** | **Enoyl-[acyl-carrier-protein] reductase** | ***Escherichia coli*** |
| 1ZBQ | - | 2.61 | -0.66 | 17-beta-hydroxysteroid dehydrogenase 4 | *Homo sapiens* |
| 1ORR | - | 2.50 | -0.33 | Cdp-tyvelose-2-epimerase | *Salmonella typhi* |
| 1GUZ | - | 2.49 | -0.62 | Malate dehydrogenase | *Chlorobium vibrioforme, chlorobium tepi* |
| 1LDN | - | 2.47 | -0.51 | L-lactate dehydrogenase | *Bacillus stearothermophilus* |
| 1U3U | - | 2.45 | -0.53 | Alcohol dehydrogenase beta chain | *Homo sapiens* |
| 2HSD | - | 2.40 | -0.55 | 3-alpha, 20 beta-hydroxysteroid dehydrogenase | *Streptomyces exfoliatus* |
| 1HEX | - | 2.39 | -1.10 | 3-isopropylmalate dehydrogenase | *Thermus thermophilus* |
| 1SC6 | - | 2.38 | -0.56 | D-3-phosphoglycerate dehydrogenase | *Escherichia coli* |
| 1HSO | - | 2.36 | -0.46 | Class i alcohol dehydrogenase 1, alpha subunit | *Homo sapiens* |
| 1D1T | - | 2.34 | -0.55 | Alcohol dehydrogenase class iv sigma chain | *Homo sapiens* |
| 2D1Y | - | 2.34 | -1.47 | Hypothetical protein tt0321 | *Thermus thermophilus* |
| 1PL8 | - | 2.28 | -0.69 | Human sorbitol dehydrogenase | *Homo sapiens* |
| 2G76 | - | 2.28 | -0.69 | D-3-phosphoglycerate dehydrogenase | *Homo sapiens* |
| 1Z2I | - | 2.23 | -1.44 | Malate dehydrogenase | *Agrobacterium tumefaciens* |
| 2G5C | - | 2.16 | -0.88 | Prephenate dehydrogenase | *Aquifex aeolicus vf5* |
| 1U3W | - | 2.10 | -0.39 | Alcohol dehydrogenase gamma chain | *Homo sapiens* |
| 1XAH | - | 2.06 | -0.48 | 3-dehydroquinate synthase | *Staphylococcus aureus* |
| 1X14 | - | 2.02 | -0.35 | Nad(p) transhydrogenase subunit alpha | *Escherichia coli* |
| 2DC1 | - | 1.99 | -0.50 | L-aspartate dehydrogenase | *Archaeoglobus fulgidus* |
| 1GV0 | - | 1.87 | -0.77 | Malate dehydrogenase | *Chlorobium tepidum* |
| 1Y3I | - | 1.86 | -0.94 | Inorganic polyphosphate/atp-nad kinase | *Mycobacterium tuberculosis* |
| 2CZC | - | 1.80 | -0.60 | Glyceraldehyde-3-phosphate dehydrogenase | *Pyrococcus horikoshii* |
| 3GPD | - | 1.74 | -1.20 | D-glyceraldehyde-3-phosphate dehydrogenase | *Homo sapiens* |
| 1QAY | - | 1.74 | -0.67 | Protein (3-hydroxy-3-methylglutaryl-coenzyme a reductase) | *Pseudomonas mevalonii* |
| 2EWM | - | 1.65 | -0.50 | (s)-1-phenylethanol dehydrogenase | *Azoarcus* |
| 1FK8 | - | 1.65 | -0.72 | 3alpha-hydroxysteroid dehydrogenase/carbonyl reductase | *Comamonas testosteroni* |
| 1EZ4 | - | 1.62 | -0.95 | Lactate dehydrogenase | *Lactobacillus pentosus* |
| 2BKJ | - | 1.56 | -0.70 | Flavin reductase | *Vibrio harveyi* |
| 1LDM | - | 1.42 | -2.36 | N/a | *N/a* |
| 2GAG | - | 1.36 | -0.57 | Heterotetrameric sarcosine oxidase alpha-subunit | *Xanthomonas maltophilia* |
| 3LDH | - | 1.29 | -0.71 | N/a | *N/a* |
| 2I9P | - | 1.23 | -0.72 | 3-hydroxyisobutyrate dehydrogenase | *Homo sapiens* |
| 1KQN | - | 0.46 | -0.55 | Nicotinamide mononucleotide adenylyl transferase | *Homo sapiens* |
| 2A9K | - | 0.44 | -0.42 | Ras-related protein ral-a | *Homo sapiens* |
| 1U1I | - | -1.16 | -3.88 | Myo-inositol-1-phosphate synthase | *Archaeoglobus fulgidus dsm 4304* |
| 1X7D | - | -6.15 | -15.35 | Ornithine cyclodeaminase | *Pseudomonas putida* |
| 1VKO | - | -13.11 | -19.53 | Inositol-3-phosphate synthase | *Caenorhabditis elegans* |
| 1OWB | - | - | - | Citrate synthase | *Escherichia coli* |
