# Supplementary material for: Drug Discovery Using Chemical Systems Biology: Repositioning the Safe Medicine Comtan to Treat Multi-Drug and Extensively Drug Resistant Tuberculosis
Source: PLoS Comput Biol. 2009 Jul 3;5(7):e1000423. doi: 10.1371/journal.pcbi.1000423 (PMC2699117; doi:10.1371/journal.pcbi.1000423)
Supplement: Table S3 — 2D small molecule similarity between existing and potential InhA inhibitors (0.06 MB DOC) [file pcbi.1000423.s008.doc]

**Drug Discovery Using Chemical Systems Biology: Repositioning the safe medicine Comtan to treat multi-drug and extensively drug resistant tuberculosis**

Sarah L. Kinnings, Nina Liu, Nancy Buchmeier, Peter J. Tonge, Lei Xie, and Philip E. Bourne

**Table S3 -** **2D small molecule similarity between existing and potential InhA inhibitors**

The 2D similarity scores (Tanimoto coefficients) of 22 InhA inhibitors to A) entacapone and B) tolcapone are shown. Their corresponding p-values were calculated from two separate density distributions of 15,000 background scores

| **A** | B |
| --- | --- |
| | **InhA Inhibitor** | **Similarity to entacapone** | **P-value** | | --- | --- | --- | | AYM | 0.155 | 0.065 | | IDN | 0.132 | 0.173 | | 566 | 0.131 | 0.184 | | 665 | 0.130 | 0.190 | | 641 | 0.128 | 0.209 | | 468 | 0.123 | 0.265 | | 744 | 0.123 | 0.265 | | ZAM | 0.123 | 0.265 | | 826 | 0.117 | 0.339 | | GEQ | 0.105 | 0.510 | | JP1 | 0.096 | 0.629 | | TN3 | 0.083 | 0.782 | | TN2 | 0.072 | 0.876 | | 654 | 0.056 | 0.959 | | TCC | 0.053 | 0.969 | | 5PP | 0.052 | 0.971 | | 8PS | 0.051 | 0.974 | | JPA | 0.048 | 0.979 | | TN5 | 0.045 | 0.984 | | TCL | 0.034 | 0.995 | | DCN | 0.028 | 0.999 | | TCT | 0.028 | 0.999 | | | **InhA inhibitor** | **Similarity to tolcapone** | **P-value** | | --- | --- | --- | | ZAM | 0.173 | 0.205 | | 826 | 0.172 | 0.211 | | 5PP | 0.162 | 0.302 | | 8PS | 0.161 | 0.316 | | AYM | 0.159 | 0.337 | | GEQ | 0.158 | 0.344 | | TN3 | 0.149 | 0.456 | | IDN | 0.148 | 0.464 | | 468 | 0.147 | 0.481 | | 744 | 0.147 | 0.481 | | 654 | 0.140 | 0.568 | | 641 | 0.114 | 0.832 | | 566 | 0.109 | 0.869 | | JPA | 0.107 | 0.882 | | TN2 | 0.104 | 0.898 | | 665 | 0.103 | 0.905 | | JP1 | 0.089 | 0.958 | | TN5 | 0.079 | 0.977 | | TCL | 0.076 | 0.982 | | DCN | 0.065 | 0.992 | | TCT | 0.064 | 0.992 | | TCC | 0.059 | 0.995 | |
