# Supplementary material for: Drug Discovery Using Chemical Systems Biology: Repositioning the Safe Medicine Comtan to Treat Multi-Drug and Extensively Drug Resistant Tuberculosis
Source: PLoS Comput Biol. 2009 Jul 3;5(7):e1000423. doi: 10.1371/journal.pcbi.1000423 (PMC2699117; doi:10.1371/journal.pcbi.1000423)
Supplement: Table S4 — Docking existing and potential InhA inhibitors onto InhA and COMT (0.05 MB DOC) [file pcbi.1000423.s009.doc]

**Drug Discovery Using Chemical Systems Biology: Repositioning the safe medicine Comtan to treat multi-drug and extensively drug resistant tuberculosis**

Sarah L. Kinnings, Nina Liu, Nancy Buchmeier, Peter J. Tonge, Lei Xie, and Philip E. Bourne

**Table S4 - Docking existing and potential InhA inhibitors onto InhA and COMT**

The results of the Surflex docking studies are shown. The mean and standard deviation of the docking scores of each molecule with nine different InhAs are given, and docking scores with COMT are included as a comparison.

| **InhA inhibitor (Ligand ID)** | **Structure** | **Docking score with InhA** | **Docking score with COMT** |
| --- | --- | --- | --- |
| 468 | 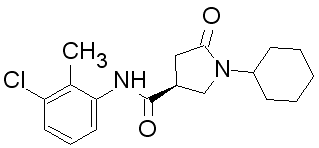 | 5.93+/-1.72 | 3.76 |
| 566 | 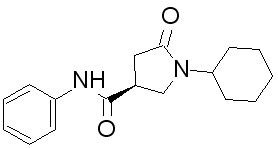 | 5.15+/-1.00 | 3.19 |
| 641 | 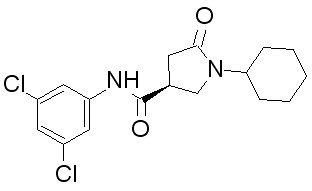 | 4.56+/-1.05 | 2.50 |
| 665 | 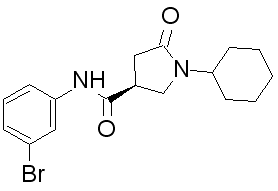 | 5.54+/-0.88 | 3.11 |
| 744 | 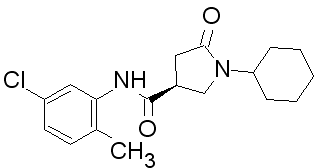 | 5.24+/-1.51 | 3.35 |
| 5PP | 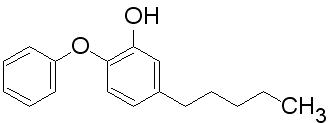 | 5.67+/-0.66 | 3.78 |
| 8PS | 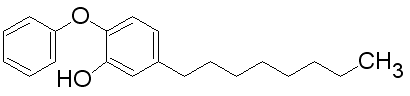 | 6.85+/-0.55 | 4.13 |
| GEQ | 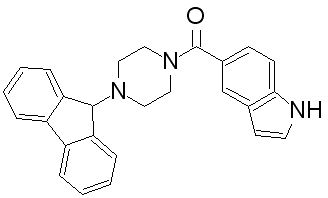 | 1.48+/-1.99 | 0.33 |
| Triclosan | 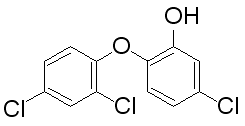 | 3.86+/-0.66 | 2.66 |
| Entacapone | 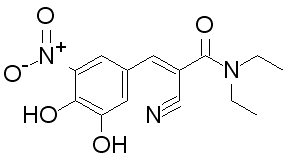 | 4.10+/-1.19 | 3.31 |
| Tolcapone | 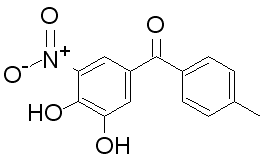 | 5.29+/-0.85 | 4.13 |
