# Supplementary material for: Drug Discovery Using Chemical Systems Biology: Repositioning the Safe Medicine Comtan to Treat Multi-Drug and Extensively Drug Resistant Tuberculosis
Source: PLoS Comput Biol. 2009 Jul 3;5(7):e1000423. doi: 10.1371/journal.pcbi.1000423 (PMC2699117; doi:10.1371/journal.pcbi.1000423)
Supplement: Table S5 — Comparison of logP and logD values between existing and potential antitubercular drugs (0.04 MB DOC) [file pcbi.1000423.s010.doc]

**Drug Discovery Using Chemical Systems Biology: Repositioning the safe medicine Comtan to treat multi-drug and extensively drug resistant tuberculosis**

Sarah L. Kinnings, Nina Liu, Nancy Buchmeier, Peter J. Tonge, Lei Xie, and Philip E. Bourne

**Table S5. Comparison of logP and logD values between existing and potential antitubercular drugs**Values were calculated using ChemSilico Predict.

| **Drug** | | **logP** | **logD**  **(pH 2)** | **logD**  **(pH 5)** | **logD**  **(pH 7.4)** |
| --- | --- | --- | --- | --- | --- |
| COMT Inhibitor | Tolcapone | 3.34 | 3.33 | 1.70 | -0.62 |
|  | Entacapone | 1.61 | 1.60 | 0.54 | -0.19 |
| First-line antitubercular drug | Ethambutol | -1.23 | -2.10 | -2.10 | -2.07 |
|  | Isoniazid | -0.72 | -1.42 | -0.73 | -0.72 |
|  | Pyrazinamide | -0.82 | -0.82 | -0.82 | -0.82 |
|  | Rifampicin | 0.97 | -2.78 | -2.78 | -2.64 |
|  | Streptomycin | -4.00 | -4.00 | -4.00 | -4.00 |
| Second-line antitubercular drug | Ciprofloxacin | -0.39 | -1.00 | -1.01 | -1.94 |
|  | Moxifloxacin | -0.07 | -1.70 | -0.81 | -1.00 |
|  | Aminosalicylic acid | 0.82 | -0.02 | -0.26 | -1.91 |
